# Genome-wide RNAi screen reveals ALK1 mediates LDL uptake and transcytosis in endothelial cells

**DOI:** 10.1038/ncomms13516

**Published:** 2016-11-21

**Authors:** Jan R. Kraehling, John H. Chidlow, Chitra Rajagopal, Michael G. Sugiyama, Joseph W. Fowler, Monica Y. Lee, Xinbo Zhang, Cristina M. Ramírez, Eon Joo Park, Bo Tao, Keyang Chen, Leena Kuruvilla, Bruno Larriveé, Ewa Folta-Stogniew, Roxana Ola, Noemi Rotllan, Wenping Zhou, Michael W. Nagle, Joachim Herz, Kevin Jon Williams, Anne Eichmann, Warren L. Lee, Carlos Fernández-Hernando, William C. Sessa

**Affiliations:** 1 Department of Pharmacology, Yale University School of Medicine, New Haven, Connecticut 06520, USA; 2 Vascular Biology and Therapeutics Program (VBT), Yale University School of Medicine, New Haven, Connecticut 06520, USA; 3 Keenan Research Centre for Biomedical Science, St. Michael's Hospital, Toronto, Ontario, Canada M5B 1W8; 4 Department of Laboratory Medicine and Pathobiology, University of Toronto, Toronto, Ontario, Canada M5S 1A8; 5 Department of Comparative Medicine, Yale University School of Medicine, New Haven, Connecticut 06520, USA; 6 Division of Endocrinology, Department of Medicine, Temple University School of Medicine, Philadelphia, Pennsylvania 19140, USA; 7 Cardiovascular Research Center, Section of Cardiovascular Medicine, Department of Internal Medicine, Yale University School of Medicine, New Haven, Connecticut 06511, USA; 8 W.M. Keck Biotechnology Resource Laboratory, Yale University School of Medicine, New Haven, Connecticut 06511, USA; 9 Human Genetics & Computational Biomedicine, Pfizer Worldwide Research and Development, Cambridge, Massachusetts 02139, USA; 10 Departments of Molecular Genetics, Neuroscience, Neurology and Neurotherapeutics, University of Texas Southwestern Medical Center, Dallas, Texas 75390, USA; 11 Department of Molecular and Clinical Medicine, Sahlgrenska Academy of the University of Gothenburg, Göteborg 41345, Sweden; 12 Departments of Biochemistry and Medicine, University of Toronto, Toronto, Ontario, Canada M5S 1A8

## Abstract

In humans and animals lacking functional LDL receptor (LDLR), LDL from plasma still readily traverses the endothelium. To identify the pathways of LDL uptake, a genome-wide RNAi screen was performed in endothelial cells and cross-referenced with GWAS-data sets. Here we show that the activin-like kinase 1 (ALK1) mediates LDL uptake into endothelial cells. ALK1 binds LDL with lower affinity than LDLR and saturates only at hypercholesterolemic concentrations. ALK1 mediates uptake of LDL into endothelial cells via an unusual endocytic pathway that diverts the ligand from lysosomal degradation and promotes LDL transcytosis. The endothelium-specific genetic ablation of Alk1 in *Ldlr-*KO animals leads to less LDL uptake into the aortic endothelium, showing its physiological role in endothelial lipoprotein metabolism. In summary, identification of pathways mediating LDLR-independent uptake of LDL may provide unique opportunities to block the initiation of LDL accumulation in the vessel wall or augment hepatic LDLR-dependent clearance of LDL.

## Abstract (web summary)

Atherosclerosis is caused by low-density lipoprotein (LDL) buildup in the vessel wall, a process thought to be mediated by LDL receptor alone. Here, the authors show that the endothelium can uptake LDL via ALK1, a TGFβ signalling receptor, suggesting new therapies for blocking LDL accumulation in the vessel wall.

## Graphical abstract

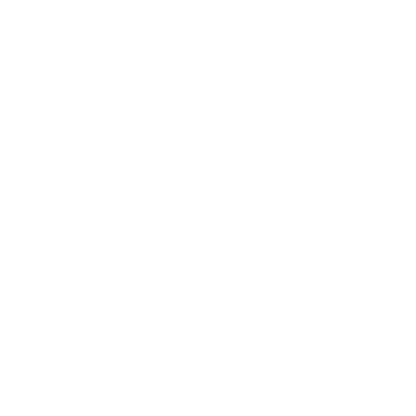

Atherosclerotic cardiovascular disease is the leading cause of death worldwide. The development of atherosclerosis is triggered by the subendothelial retention of plasma-derived apoB-lipoproteins, particularly LDL and apolipoprotein-B (apoB)-containing remnants^1^,^2^,^3^,^4^. The most effective therapy to date that reduces atherosclerotic cardiovascular disease, namely lowering plasma LDL levels, works by decreasing circulating apoB containing lipoprotein levels, thereby reducing the likelihood that these particles will enter and become retained within the arterial wall. Despite this well appreciated series of events leading to disease and effective therapies lowering plasma LDL levels, the molecular mechanisms of how LDL is transported into and across the endothelium have not been elucidated.

LDL in the blood has to enter the endothelium and cross it to reach the subendothelial area, where it is retained and accumulates over time. Electron microscopy studies have demonstrated that physiological levels of LDL particles can be internalized by two pathways; an LDL receptor (LDLR) dependent and LDLR independent pathways^5^. The LDLR-mediated pathway promotes LDL degradation and is downregulated at the higher concentrations of LDL^6^ while the latter pathway is enhanced with hypercholesterolemic concentrations of LDL^5^. Additional studies have shown that at least 50% of the LDL that is endocytosed by the endothelium traverses the cells to reach the basolateral side via an unknown active transport mechanism^7^,^8^ and an LDLR independent initial route of LDL permeation into the artery wall has been previously described^9^. A recent paper has described that reducing ApoB containing lipoproteins in an established model of atherosclerosis rapidly reduces LDL permeation into the vessel wall. Interestingly the flux of LDL is separable from the total content of LDL in vessel wall and this improved barrier function precedes plaque regression^10^. Thus, the regulated entry of LDL into the vessel wall is essential for both the formation and regression of atheromas.

With this background in mind, we sought to elucidate the genes required for native LDL uptake into endothelial cells using a genome-wide RNAi approach to target over 18,000 genes followed by high content confocal imaging of fluorescent DiI-LDL uptake. Primary gene hits were further analysed in secondary screens to assess broad effects on endocytosis and sterol sensing permitting the identification of 34 high confidence hits, three of which are unique to the endothelium. The gene hits were also cross-referenced against publically available genome-wide association studies (GWAS). Here we characterize one of these hits in detail, *ACVRL1* (also called ALK1) which fulfils the criteria for a novel low-affinity, high-capacity receptor for LDL in endothelial cells that functions during hypercholesterolemia and promotes LDL transcytosis.

## Results

### Genome-wide RNAi screen in endothelial cells

**Figure 1:**
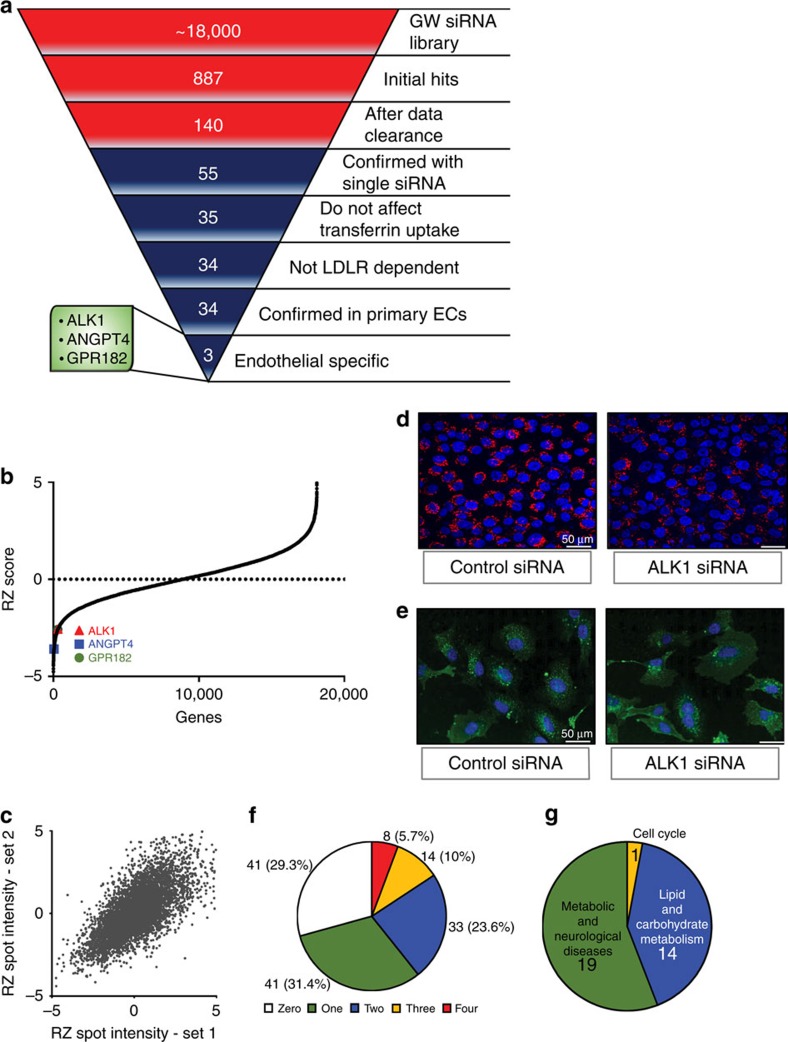
Screen to identify pathways regulation LDL uptake. (**a**) Summary of the results from the RNAi screen. Results from genome-wide RNAi screen are red while follow-up screens are in blue. (**b**) Robust z-score-scores from the original screen. The inverse sigmoidal robust z-score-score distribution of genes indicates that genes can either increase or decrease DiI-LDL uptake and (**c**) shows the reproducibility between two individual sets of plates from the screen. (**d**) Representative image (scale bar, 50 μm.) of a gene knockdown of ALK1 resulting in less DiI-LDL uptake, whereas the loss of ALK1 (**e**) did not affect the uptake of FITC transferrin (scale bar, 50 μm). (**f**) Pie chart of 140 hits tested for DiI-LDL uptake inhibition in the follow-up screen, showing the number (and percentage) of active siRNAs for each hit. (**g**) Pathway clustering of the final 34 hits.

The uptake, transfer and retention of LDL particles across the endothelial layer of blood vessels is considered a primary mechanism to initiate atherogenesis. However, since the LDLR is typically occupied and downregulated when plasma lipids are elevated, we undertook a genome-wide RNAi screen to identify genes involved in native LDL uptake independent of LDLR activity. Considering the importance of genetic stability and reproducibility required for a screen of this calibre, the human endothelial cell line, EA.hy926 (ref. 11) (Fig. 1a) was used and cultured under conditions where endogenous LDLR had been downregulated by excess of the ligand LDL^6^. In the initial screen, run in triplicate over a 3 months period, cells were transfected with a Dharmacon short interfering RNA (siRNA) library containing four pooled siRNAs/gene to silence 18,119 genes in the human genome (Supplementary Data set 1). Transfected cells were then incubated with excess human LDL (25 μg ml^−1^) overnight to downregulate LDLR overnight, before the uptake of fluorescently labelled LDL (DiI-LDL) was examined after 60 min using a 384 well confocal microscope. The results from the screen were fit to an expected inverse sigmoidal robust z-score distribution (Fig. 1b), indicating that gene knockdown either increased or decreased DiI-LDL uptake and demonstrated a high level of reproducibility between different data sets (Fig. 1c). As seen in Fig. 1a, silencing of 887 genes showed an effect on DiI-LDL uptake with a robust z-score ≤−2.5. A manual, computer-assisted data clearance algorithm removed promiscuous genes (that typically show up in various screens), toxic genes, and artefacts by visual inspection of the confocal images from individual hits. The data were mined to include cell surface molecules and novel gene products, but to exclude genes for transcription factors, obvious components of the endocytic machinery and sterol regulated genes. After inspection of individual hits, a final set of 140 genes (Supplementary Data set 2) was re-screened using four individual siRNAs per gene resulting in the confirmation of 55 genes (with ≥2 siRNAs/gene showing ≥50% reduction of DiI-LDL uptake) required for DiI-LDL uptake (Fig. 1d,f). To identify pathways specific for LDL and not classical cargo molecules, a secondary screen examining the uptake of transferrin-fluorescein isothiocyanate (FITC), a marker for clathrin-mediated endocytosis was performed. The silencing of 35/55 genes did not affect the uptake of transferrin (Fig. 1e). Finally, the contribution of LDLR, in conjunction with the newly identified genes, was tested using cells stably expressing short hairpin RNAs (shRNAs) against *LDLR* (Supplementary Fig. 1) for *LDLR* messenger RNA and protein levels) and 34 of these genes reduced DiI-LDL uptake independent of LDLR levels. Furthermore, since the original screen was conducted in an endothelial line, the 34 hits identified were retested in primary cultures of human umbilical vein endothelial cells (HUVEC) and all 34 hits were re-confirmed. Analysis of the 34 genes with Ingenuity Pathway Analysis (Fig. 1g, Supplementary Fig. 2 and Supplementary Table 1) showed that 19 hits cluster in metabolic/neurological pathways and 14 belong to lipid/carbohydrate metabolic pathways and only three genes were uniquely expressed in endothelial cells. Analysis of publically available GWAS-data sets revealed an association for 14 gene hits in regard to cardiovascular traits and/or lipids (Supplementary Fig. 3 and Supplementary Data set 3). *ACVRL1, ANGPT4* and *GPR182* fulfilled all the criteria of the follow-up screen (Fig. 1a and Supplementary Fig. 3). Since ANGPT4 is not well characterized as a ligand and GPR182 is an orphan receptor, the initial follow-up focuses on ALK1 as an LDL-binding protein mediating LDL uptake and transcytosis.

### Specificity of ALK1 deficiency for apoB containing lipoproteins

**Figure 2:**
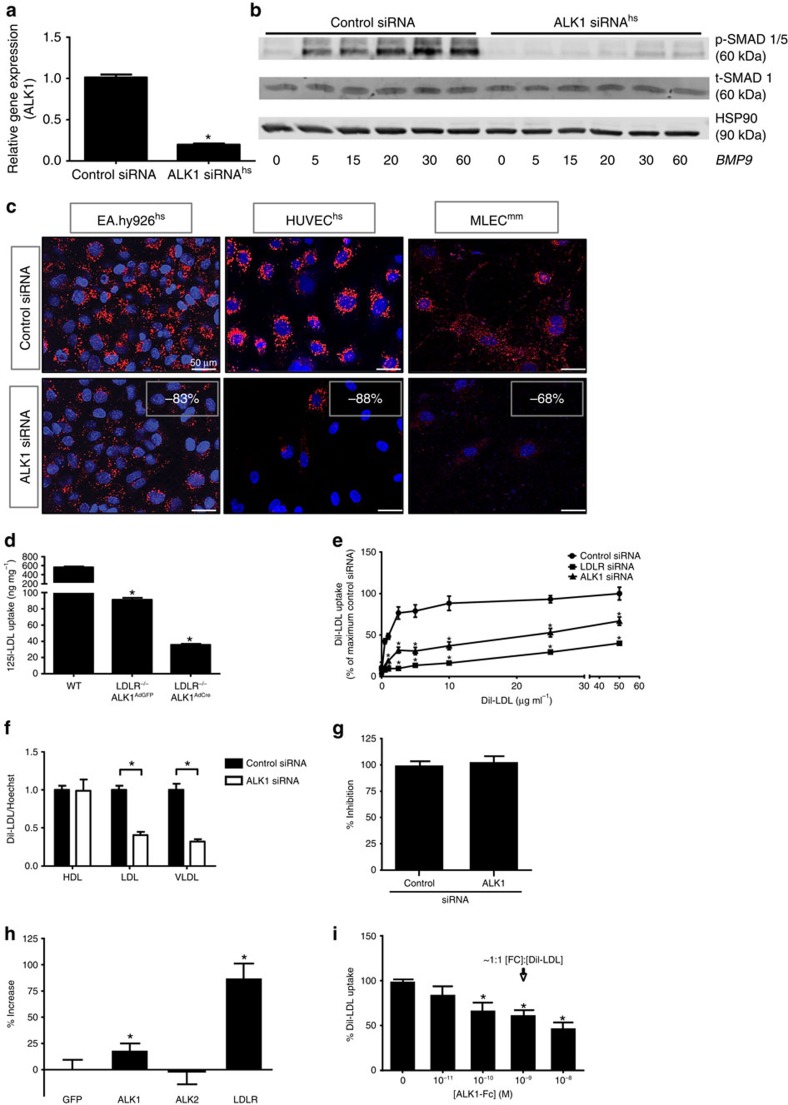
Validation of ALK1 in mediating LDL uptake into endothelium. (**a**) Quantitative PCR analysis for the knockdown efficiency of the ALK1 siRNA in human endothelial cells (HUVEC). Data represent the mean±s.e.m. and are representative of three experiments in duplicate. *P<0.05, Student's *t*-test. (**b**) Western blot analysis showing the knockdown efficiency of the *ACVRL1* siRNA in HUVEC based on the BMP9 (10 ng ml^−1^) induced phosphorylation of canonical SMAD 1/5 phosphorylation. A non-cropped western blot for this experiment can be found in Supplementary Fig. 9a. (**c**) DiI-LDL uptake was reduced in various human (EA.hy926 and HUVEC) and mouse (MLEC) endothelial cells treated with *ACVRL1*/*Acvrl1* siRNA. Scale bar, 50 μm. (**d**) ^125^I-LDL uptake into WT and *Acvrl1*^fl/fl^/*Ldlr*^*−/−*^ MLEC. *Acvrl1*^fl/fl^/*Ldlr*^*−/−*^ MLEC were infected with AdGFP (control) or AdCre/GFP, then the uptake (includes bound LDL) of ^125^I-LDL was examined. Cells were kept in LPDS overnight before uptake studies. Data represent the mean±s.e.m. and are representative of three experiments. *P<0.05, Student's *t*-test. (**e**) Cells were treated with control siRNA, *LDLR* siRNA or *ACVRL1* siRNA and placed into regular media, washed and exposed to increasing concentrations of DiI-LDL. Data represent the mean±s.e.m. and are representative of three experiments. *P<0.05, Student's *t*-test. (**f**) Uptake analysis of DiI-HDL, -LDL and -VLDL (2.5 μg ml^−1^) into endothelial cells treated with control siRNA and *ACVRL1* siRNA. Data represent the mean±s.e.m. and are representative of three experiments. *P<0.05, Student's *t*-test. (**g**) Uptake of oxidized LDL (2.5 μg ml^−1^) into EA.hy926 cells treated with control siRNA and *ACVRL1* siRNA cultured overnight in LPDS media supplemented with 25 μg ml^−1^ LDL. Data represent the mean±s.e.m. and are representative of three experiments. (**h**) Comparison of DiI-LDL uptake into *Ldlr*-KO MEFs transfected with either GFP (negative control), ALK1-GFP, ALK2-GFP or LDLR-GFP (positive control). Data represent the mean±s.e.m. and are representative of three experiments in duplicates. *P<0.05, Student's *t*-test. (**i**) Uptake of DiI-LDL in EA.hy926 cells in the presence of increasing concentrations of ALK1-Fc was measured. Addition of 10^−9^ M ALK1-Fc is equimolar to DiI-LDL (500 ng ml^−1^). Data represent the mean±s.e.m. and are representative of three experiments. *P<0.05, Student's *t*-test.

ALK1 is a TGF-β-type 1 receptor that binds bone morphogenetic proteins (BMP) −9 and −10 ligands with high affinity^12^. The receptor is highly expressed in primary human endothelial cells compared primary human hepatocytes (Supplementary Fig. 4). To examine how this receptor may regulate LDL uptake, in depth analysis of ALK1 was undertaken in a variety of systems. Knockdown of ALK1 reduced transcript levels in human endothelial cells (Fig. 2a) and mouse lung endothelial cells (MLEC; Supplementary Fig. 5a). All four individual siRNAs against human ALK1 from the genome-wide RNAi screen were analysed for their knockdown efficiency, showing that siRNA 06 led to the strongest inhibition (Supplementary Fig. 5b). Since several commercially available antibodies do not detect ALK1 protein specifically, we used BMP9 signalling to SMAD1/5 as a surrogate readout for the loss of ALK1 function. Indeed, knockdown of ALK1 impaired BMP9 induction of canonical SMAD 1/5 phosphorylation in HUVECs (Fig. 2b) and MLEC (Supplementary Fig. 5c). Moreover, ALK1 silencing in EA.hy926 cells, primary HUVEC and MLEC resulted in reduced uptake of DiI-LDL (Fig. 2c). To test the sufficiency of ALK1 for LDL uptake, MLEC were isolated from *Acvrl1*^*fl/fl*^*, Ldlr*^*−/−*^ double knockout (KO) mice, immortalized with middle T antigen^13^ cultured in lipoprotein-deficient serum (LPDS) overnight to maximize LDL uptake and infected with adenovirus-expressing green fluorescent protein (AdGFP) as a control or adenoviral Cre-recombinase (AdCre) to excise the *Acvrl1* allele. The loss of LDLR markedly reduced ^125^I-LDL uptake into MLEC infected with AdGFP consistent with its known role of LDLR (Fig. 2d), however, AdCre-mediated excision of *Acvrl1* in cells lacking LDLR further reduced ^125^I-LDL uptake by 60%. Next, we compared the relative importance of LDLR versus ALK1 in mediating LDL uptake by treating endothelial cells (EC) with siRNAs to *LDLR* or *ALK1* and growing the cells in complete media including serum. Under these conditions, the loss of LDLR and to a lesser extent ALK1, both reduced LDL uptake over a range of DiI-LDL concentrations (Fig. 2e). Also, the specificity of the uptake of ApoB100 rich lipoproteins (LDL and very low-density lipoprotein; VLDL) versus ApoA1 rich lipoproteins (HDL) was tested. Silencing of ALK1 inhibited DiI-LDL and DiI-VLDL uptake, but not DiI-HDL uptake (Fig. 2f), implying specificity for ApoB100 containing lipoproteins. Chylomicrons or its remnants were not tested. It has been hypothesized that oxidized LDL (OxLDL) contributes to the development of atherosclerosis, and it is known that the biological behaviour and receptor recognition of OxLDL is significantly different from native LDL^14^. The uptake of DiI-OxLDL is not affected by the knockdown of ALK1 (Fig. 2g). ALK1 is one of seven ALK receptors (ALK1-ALK7) and sequence alignment of this family reveals low homology in the amino-terminal/extracellular domains, but a high homology of the carboxy-terminal/intracellular domain. As the ligands bind to the amino-terminal domain of the ALK family we compared ALK1 and ALK2, which share 59% homology among the entire protein and just 23% for the extracellular domain. Whereas LDLR and ALK1 overexpression increases DiI-LDL uptake we could not detect an effect of ALK2 overexpression on DiI-LDL uptake (Fig. 2h). As the knockdown of ALK1 in endothelial cells results in less uptake of LDL, the presence of the ALK1-ectodomain (ALK1-Fc) should phenocopy this effect. ALK1-Fc reduced DiI-LDL uptake dose-dependently starting at a threshold of 10^−10^ M (Fig. 2i).

### ALK1 knockdown does not affect sterol sensing

**Figure 3:**
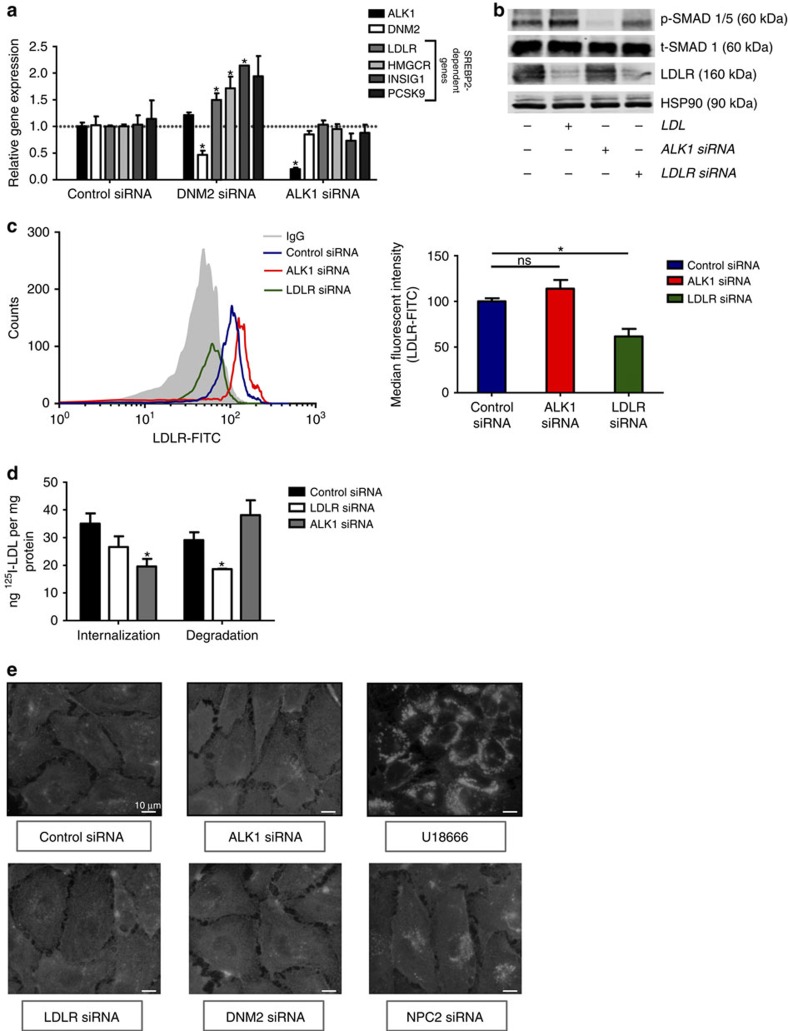
ALK1 deficiency does not affect sterol sensing in the endothelium. (**a**) Quantitative PCR analysis of SREBP2-dependent genes after knockdown of *DNM2* or *ACVRL1*. The loss of DNM2 increases SREBP2-dependent gene expression, whereas the loss of ALK1 does not. Data represent the mean±s.e.m. and are representative of three experiments in duplicates. *P<0.05, Student's *t*-test. (**b**) Western blot analysis of the BMP9 (10 ng ml^−1^) induced phosphorylation of SMAD 1/5. HUVEC were incubated in LPDS and exposed to BMP9 for 60 min. In lane 2, cells were pretreated with LDL (25 μg ml^−1^) to downregulate LDLR. In lanes 3 and 4, ALK1 or LDLR was silenced with siRNA, respectively. A non-cropped western blot for this experiment can be found in Supplementary Fig. 9b. (**c**) The loss of ALK1 does not influence LDLR on the cell surface. Flow cytometric analysis of cell surface LDLR levels in endothelial cells treated with control, *ACVRL1* or *LDLR* siRNAs. The Ab C7 was used for LDLR and IgG is an isotype control and data quantified in right panel. Data represent the mean±s.e.m. and are representative of three experiments in triplicates. *P<0.05, Student's *t*-test. (**d**) ^125^I-LDL internalization and degradation in cells treated with control, *ACVRL1* or *LDLR* siRNAs. EA.hy926 cells were pre-incubated with LDL (25 μg ml^−1^) overnight and the internalization and degradation of ^125^I-LDL was after 4 h of incubation. *ACVRL1* siRNA reduced internalization and had no effect on LDL degradation, whereas the *LDLR* siRNA (as a positive control) reduced LDL internalization and led to less degradation of LDL. Data represent the mean±s.e.m. and are representative of three experiments in duplicates. *P<0.05, Student's *t*-test. (**e**) Loss of ALK1 does not increase cellular free cholesterol. Filipin-III staining was examined in endothelial cells treated with siRNAs for *ACVRL1*, *LDLR*, *DNM2* and *NPC2* siRNA or treated with U18666 to enhance free cholesterol. Scale bar, 10 μm. Data are representative of at least four experiments. ns, not significant.

As cholesterol homeostasis is crucial for cell growth and maintenance, cholesterol uptake, synthesis and metabolism, is largely regulated through sterol regulatory element-binding protein 2 (SREBP2) of sterol responsive genes. As seen in Fig. 3a, knockdown of dynamin 2 (DNM2) in EA.hy926 cells grown in complete media and serum, did not influence *ACVRL1* gene expression but upregulated the transcript levels of several *SREBP2*-dependent genes (*LDLR*, *HMGCR*, *INSIG1* and *PCSK9*), whereas the loss of *ACVRL1* did not affect *SREBP2*-dependent gene expression. A further analysis was performed in EA.hy926 cell cultured in LPDS or LPDS containing LDL (25 μg ml^−1^, Supplementary Fig. 6). As expected, the addition of LDL to LPDS resulted in downregulation of *LDLR*, *HMGCR* and *INSIG1*—all *SREBP2*-dependent genes. Importantly, the loss of *DNM2*, but not *ACVRL1*, blunted the ability of LDL to reduce gene expression showing that ALK1 does not influence sterol sensing. Moreover, silencing of *ACVRL1* had no effect on the total levels of LDLR in cell lysates by western blotting (Fig. 3b) or on cell surface LDLR quantified by fluorescence-activated cell sorting (FACS) (Fig. 3c). Because the uptake of LDL by the LDLR results in its lysosomal degradation^15^, we assessed the uptake and degradation of ^125^I-LDL in cells deficient in *LDLR* or *ACVRL1*. EA.hy926 were transfected with control, *LDLR* or *ACVRL1* siRNAs, and then exposed to excess LDL (25 μg ml^−1^) overnight. The next morning, cells were incubated with ^125^I-LDL and the uptake and degradation (after 4 h) was assessed^6^. The net uptake of ^125^I-LDL in LDL pretreated EA.hy926 cells (Fig. 3d) was lower than that in MLEC cultured in LPDS, Fig. 2d). Knockdown of ALK1 in LDL pretreated EA.hy926 cells reduced ^125^I-LDL internalization, whereas knockdown of LDLR did not. However, the degradation of ^125^I-LDL, assessed by free ^125^I-tyrosine in the medium, was reduced by the loss of LDLR, but not the loss of ALK1. The data suggests that the suppression of LDLR by pre-treatment with LDL was incomplete, and so the subsequent addition of siRNA against *LDLR* further suppressed LDL internalization and degradation via the LDLR. As ALK1-mediated LDL uptake did not affect sterol sensing or LDL degradation, the pool of non-esterified, free cholesterol was analysed using Filipin-III staining (Fig. 3e). The knockdown of *ACVRL1*, *LDLR* or *DNM2* did not increase the pool of free cholesterol, whereas both positive controls (U18666 (ref. 16) and *NPC2* siRNA) did. These data demonstrate that ALK1 facilitates LDL uptake but does not target LDL for degradation.

### ALK1 increases LDL uptake independent of its kinase activity

**Figure 4:**
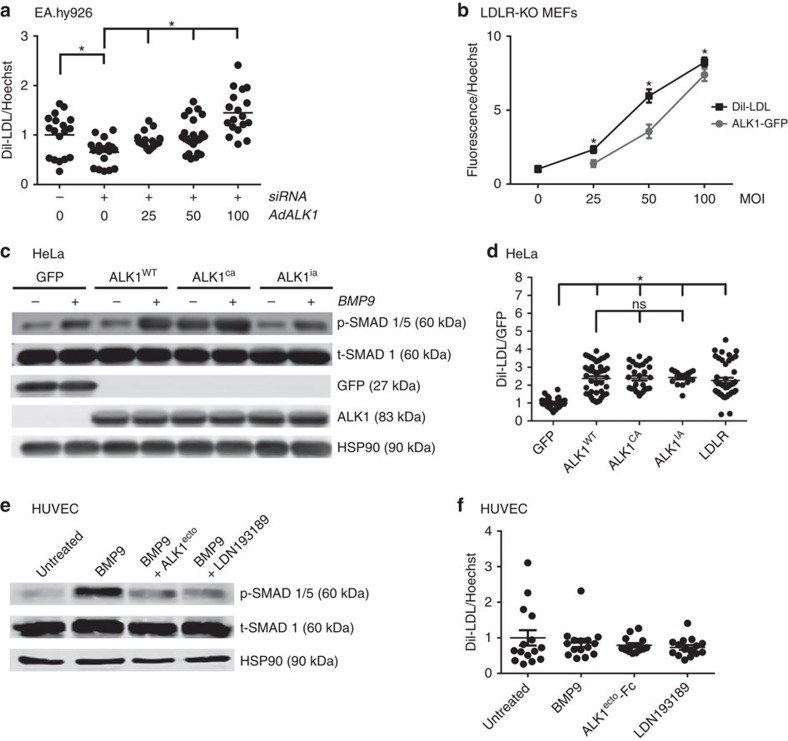
ALK1 rescues LDL uptake and promotes LDL uptake independent of its kinase activity. (**a**) Analysis of DiI-LDL uptake in EA.hy926 treated with *ACVRL1* siRNA (+) in the presence of increasing amounts of expressed ALK1 (using various MOI of AdALK1-GFP). DiI-LDL uptake was normalized by Hoechst dye stained nuclei. Data represent the mean±s.e.m. and are representative of three experiments in triplicates. *P<0.05, Student's *t*-test. (**b**) ALK1 dose dependently increases DiI-LDL uptake in *Ldlr*-KO MEFs. DiI-LDL data are normalized for ALK1-GFP expression in *Ldlr*-KO MEFs by using various MOI of AdALK1-GFP. Data represent the mean±s.e.m. and are representative of three experiments. *P<0.05, Student's *t*-test. (**c**) HeLa cells were transfected with either GFP, WT, constitutively active (CA, Q201D) and inactive variant (IA, R374Q) ALK1 constructs and p-SMAD1/5 levels were examined in the absence or presence of BMP9 (10 ng ml^−1^) (Data are mean±s.e.m., experiment was performed three times). A non-cropped western blot for this experiment can be found in Supplementary Fig. 10a. (**d**) DiI-LDL uptake analysis of cells expressing GFP and the different variants of ALK1. Data represent the mean±s.e.m. and are representative of three experiments in triplicates. *P<0.05, Student's *t*-test. (**e**) Western bot analysis of p-SMAD1/5 after starvation using BMP9 (10 ng ml^−1^) or pharmacological inhibitors (ALK1^ecto^,400 ng ml^−1^: tenfold molar excess or LDN193189, 50 nM). A non-cropped western blot for this experiment can be found in Supplementary Fig. 10b. (**f**) BMP9 stimulation or inhibition does not affect DiI-LDL uptake into endothelial cells. Data represent the mean±s.e.m. and are representative of three experiments. *P<0.05, Student's *t*-test. ns, not significant.

To examine the sufficiency of ALK1 for LDL uptake, rescue and gain-of-function experiments were performed. As shown in Fig. 4a, DiI-LDL uptake is significantly decreased in endothelial cells after knockdown of ALK1, an effect rescued, in a dose-dependent manner (0–100 MOI of adenovirus), by the expression of an adenovirus encoding ALK1-GFP (AdALK1). Moreover, infection of AdALK1-GFP into *Ldlr*-KO mouse lung fibroblasts (MEF) also dose-dependently increased DiI-LDL uptake (Fig. 4b), showing again that ALK1 mediates LDL uptake independently of the LDLR.

To examine if the kinase activity of ALK1 contributed to its ability to promote LDL uptake, GFP (negative control), wild-type (ALK^WT^), a constitutively active mutant (Q201D; ALK1^ca^) or an inactive variant (R374Q; ALK1^ia^) of ALK1 were expressed in HeLa cells, which lack ALK1. In ALK1^wt^ transfected cells, BMP9 (10 ng ml^−1^) stimulated the phosphorylation of SMAD 1/5 (Fig. 4c) an effect augmented in ALK^ca^ expressing cells and diminished in ALK^ia^ cells. Expression of all three ALK1 constructs increase DiI-LDL uptake compared with GFP in HeLa cells, but no difference among these three variants could be detected (Fig. 4d), showing that the uptake of LDL is independent of ALK1 kinase activity. As a positive control, LDLR-GFP also increased DiI-LDL uptake. Moreover, in HUVEC, activation of ALK1 with BMP9, neutralization of BMP9 with soluble ALK1 ectodomain (400 ng ml^−1^, tenfold molar excess over BMP9) or the pharmacological ALK-inhibitor (LDN193189, 50 nM) resulted in the expected effect on p-SMAD 1/5 levels (Fig. 4e), but all three conditions did not affect the uptake of DiI-LDL (Fig. 4f).

### LDL binds to ALK1 directly

**Figure 5:**
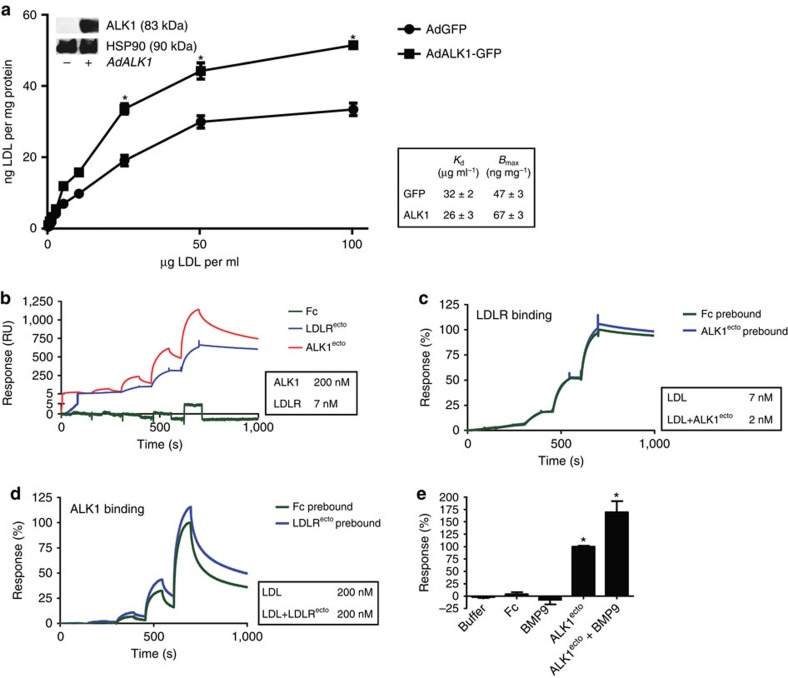
Cellular and direct binding of LDL to ALK1. (**a**) *Ldlr*-KO MEFs were infected with AdGFP or AdALK1-GFP and the concentration-dependent binding of LDL at 4 °C determined. Cells were kept in LPDS overnight. Insert shows western blot for the expression of ALK1-GFP with HSP90 as a loading control. A non-cropped western blot for this experiment can be found in Supplementary Fig. 11a. Table shows *K*_d_ and *B*_max_. Data represent the mean±s.e.m. and are representative of three experiments in triplicates. *P<0.05, Student's *t*-test. (**b**) SPR analysis of binding of ALK1 to LDL. The analytes Fc (0.1, 0.2, 0.6, 1.8, 5.4 μM), LDLR^ecto^ (5, 14, 43, 129, 388 nM) and ALK1^ecto^: (0.19, 0.57, 1.7, 5.1, 15.3 μM) were assayed for binding to immobilized LDL. Sensorgram depicts binding events from a representative experiment and table shows *K*_d_ calculated from three independent experiments. (**c**) SPR analysis of LDLR^ecto^ binding on naive or ALK1^ecto^ saturated LDL. ALK1^ecto^ (15.3 μM) was prebound to LDL and exposed to LDLR^ecto^ (5, 14, 43, 129, 388 nM). Results show no competition between ALK1 and LDLR in binding to LDL, indicating distinct binding domains on LDL for ALK1 and LDLR. Table shows *K*_d_ calculated from three independent experiments. (**d**) SPR analysis of ALK1^ecto^ binding on naive or LDLR^ecto^ saturated LDL. LDLR^ecto^ (388 nM) was prebound to LDL and exposed to ALK1^ecto^ (0.19, 0.57, 1.7, 5.1, 15.3 μM). This inverse experiment of Fig. 6c shows no competition between ALK1 and LDLR in binding to LDL and the inset shows *K*_d_ calculated from three independent experiments. (**e**) SPR analysis of binding of Fc, BMP9, ALK1^ecto^ and ALK1^ecto^/BMP9 complex to LDL (all proteins at 2 μM). This result indicates that LDL and BMP9 bind separate domains on ALK1 as ALK1 binding to LDL is not inhibited by the presence of BMP9. Data represent the mean±s.e.m. and are representative of three experiments. *P<0.05, Student's *t*-test.

To examine novel functions of ALK1 that could mediate LDL uptake, we used several approaches. First, LDLR-KO MEF were infected with adenoviral constructs expressing GFP (AdGFP) or ALK1-GFP (AdALK1-GFP) and the cell surface binding of ^125^I-LDL was examined. In ALK1-GFP transduced LDLR-KO MEF, the cell surface binding of ^125^I-LDL was enhanced in these cells by 30% to 67±3 ng LDL mg^−1^ with a *K*_d_ of 26±3 μg ml^−1^ (Fig. 5a). Next to directly examine protein–protein interactions between LDL and ALK1, surface plasmon resonance (SPR) was used with LDL immobilized on the chip and purified fragments of the ectodomains of LDLR and ALK1 were used as analytes^17^. As seen in Fig. 5b, the specific binding of LDLR^ecto^ and ALK1^ecto^ was clear, with an apparent *K*_d_ of 7 and 200 nM for binding to LDLR and ALK1, respectively. To test if LDLR and ALK1 compete for binding to LDL, immobilized LDL was pre-bound with ALK1 or LDLR ectodomains followed by the addition of the other ectodomain. But neither the binding of LDLR^ecto^ was altered when the LDL surface was pretreated with ALK1^ecto^ (Fig. 5c), nor was the binding of ALK1^ecto^ altered when the LDL surface was pretreated with LDLR^ecto^ (Fig. 5d). These results indicate that LDLR and ALK1 bind to different sites on LDL, presumably on ApoB. Finally, ALK1^ecto^ binding to LDL was analysed in the presence of equimolar concentrations of its cognate ligand BMP9. ALK1^ecto^ binding was not perturbed by the presence of BMP9 (Fig. 5e) suggesting at least two distinct sites on the extracellular domain of ALK1 for binding each protein.

### ALK1-LDL complex internalizes into perinuclear compartment

**Figure 6:**
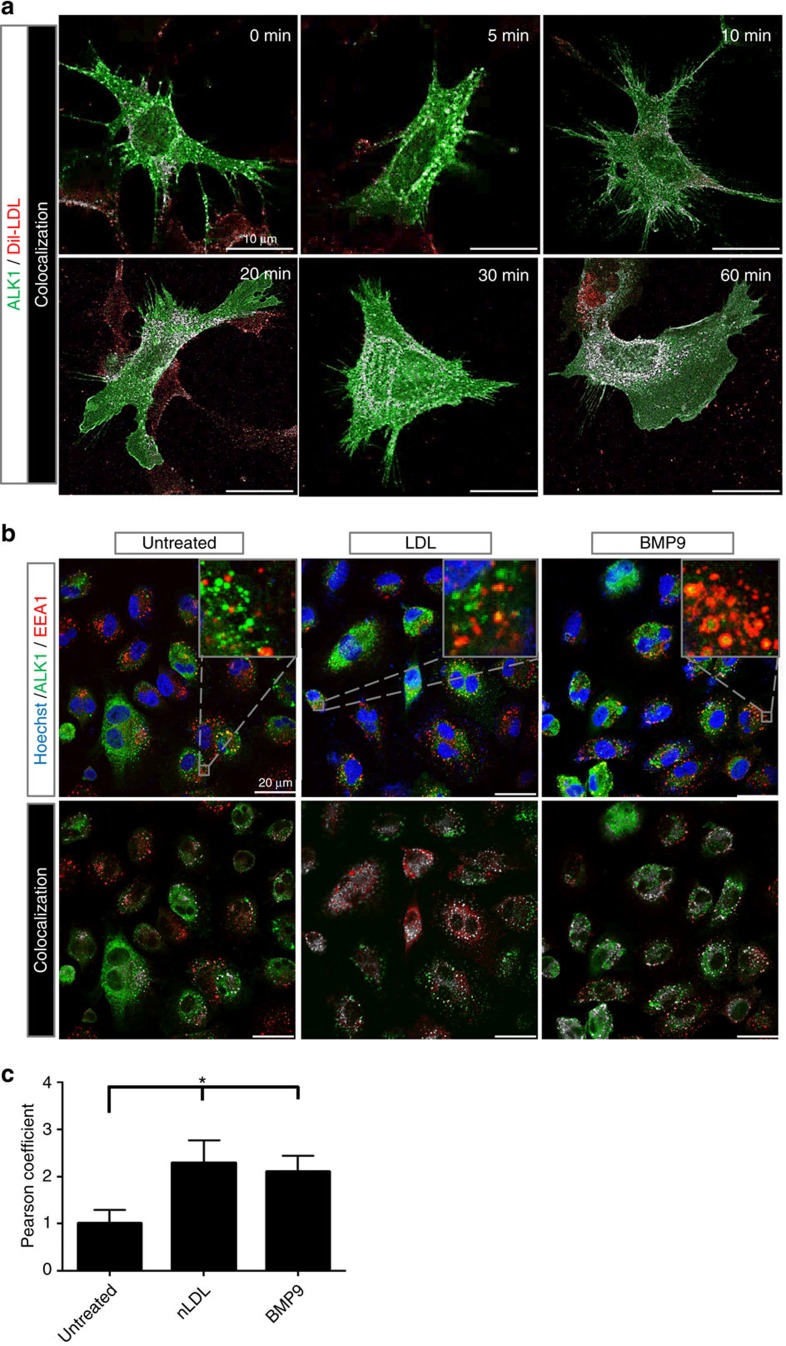
Time-dependent internalization and co-localization of DiI-LDL and ALK1. (**a**) *Ldlr*-KO MEFs were infected with ALK1-GFP, incubated in LPDS overnight and the time-dependent internalization of DiI-LDL examined at 37 °C. Cells were imaged for ALK1-GFP (green), DiI-LDL (red) and white reflecting co-localization of ALK1-GFP/DiI-LDL (Menders correlation). The internalization of DiI-LDL and its co-localization with DiI-LDL shows at 10–20 min and accumulates in a perinuclear compartment after 60 min. Scale bar, 10 μm. The data is representative for three independent experiments. (**b**) Analysis of ALK1-GFP localization in control (untreated), LDL (25 μg ml^−1^) or BMP9 (10 ng ml^−1^) treated EA.hy926 cells. Cells were infected for 48 h and transferred to LPDS for the remaining 24 h. Cells were treated as described for 1 h at 37 °C, fixed and imaged. Upper panels show original confocal laser scanning microscopy images (green, ALK1-GFP; red, EEA1; blue, nuclei). Lower panels show Menders correlation (green, ALK1-GFP alone; red, EEA1 alone; white, ALK1-GFP/EEA1 co-localized). Scale bar, 20 μm. The data is representative for three independent experiments. (**c**) Bar graph shows Pearson correlation of these three conditions. The result indicates a co-localization of ALK1 with the early endosome marker EEA1 upon stimulation with either LDL or BMP9 within 1 h. Data represent the mean±s.e.m. and are representative of three experiments. *P<0.05, Student's *t*-test.

To examine if the binding of LDL to ALK1 influences its internalization, imaging experiments were performed in *Ldlr*-KO MEFs infected with AdALK1-GFP and incubated with DiI-LDL from 0–60 min. As seen in Fig. 6a, ALK1-GFP co-localizes in a time-dependent manner with DiI-LDL in the cell with a perinuclear accumulation at 60 min. At 60 min, ALK1-GFP localized in an early endosomal compartment marked by EEA1 (Fig. 6b). Analysis of the images using Pearson correlation (Fig. 6c) showed a significant increase in co-localization of ALK1-GFP and EEA1 after stimulation with either LDL or BMP9.

### ALK1 mediates LDL transcytosis

**Figure 7:**
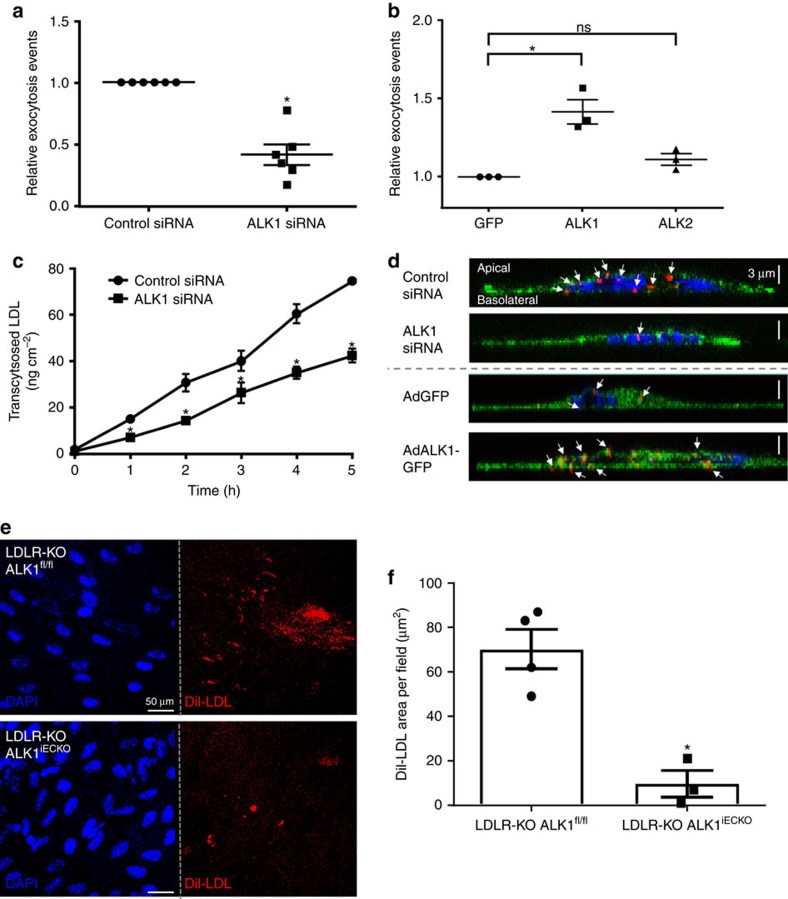
ALK1 mediates transcytosis and *in vivo* uptake of LDL. (**a**) HCAECs were treated with PCSK9 to remove LDLR and transfected with either control siRNA or *ACVRL1* siRNA. TIRF-based transcytosis assay was performed to measure the effect of ALK1 on LDL transcytosis. Each data point represents over five individual cells measured in six independent experiments. *P<0.05, Student's *t*-test. (**b**) PCSK9 treated HCAECs were transfected with GFP, ALK1 or ALK2 to measure transcytosis using TIRF imaging. Each data point represents over 15 individual cells measured in three independent experiments. *P<0.05, Student's *t*-test. (**c**) Transwell assay for LDL transcytosis in HCAECs incubated with ^125^I-labelled LDL. Data represent the mean±s.e.m. and are representative of three independent experiments with two batches of ^125^I-LDL. *P<0.05, Student's *t*-test (**d**) Cross-section imaging of EA.hy926 cells transfected with either control siRNA or ALK1 siRNA or infected with adenovirus encoding GFP or ALK1-GFP (blue, nucleus; green, top panel: lectin/bottom panel: GFP; red, DiI-LDL). The data is representative for three independent experiments. (**e**) Representative *en face* images of DiI-LDL uptake into the inner curvature of the aortic arch of *Ldlr*-KO animals with or without endothelial expression of *Acvrl1* (blue, nucleus; red, DiI-LDL). Scale bar, 50 μm. (**f**) Quantification of DiI-LDL uptake into the inner curvature endothelium of the aortic arch *Ldlr*-KO animals with or without endothelial expression of ALK1. Data represent the mean±s.e.m. and are from 4 and 3 mice, respectively. *P<0.05, Student's *t*-test. ns, not significant.

Collectively, the data suggests that LDL uptake can be mediated by the direct binding of LDL to ALK1 followed by its internalization via a non-degradative pathway raising the question if ALK1 can mediate LDL transcytosis across the endothelium. Recently, a novel method to assess LDL transcytosis using total internal reflectance microscopy (TIRF) was developed^18^ where the docking, fusion and transcytosis of LDL could be easily quantified in human coronary arterial endothelial cells (HCAEC) treated with PCSK9 to remove LDLR from the surface (see Supplementary Fig. 7). Using this assay, knockdown of ALK1 leads to significantly reduced transport of DiI-LDL from the apical to the basolateral membrane (Fig. 7a and Supplementary Movies 1 and 2), indicating that ALK1 mediates LDL transcytosis. Moreover, over-expression of ALK1, but not ALK2, increases LDL transcytosis (Fig. 7b). To complement the TIRF analysis, the transport of ^125^I-LDL through a confluent layer of HCAEC cultured on transwell chambers (0.4 μm) confirmed the effect of ALK1 on LDL transcytosis (Fig. 7c). Finally, 3D reconstruction of Z stacks of EA.hy926 cells transfected with control siRNA or *ACVRL1* siRNA (Fig. 7d, top panel) demonstrate less DiI-LDL particles near the basolateral membrane (indicated by white arrows) in cells transfected with ALK1 siRNA and overexpression of ALK1 using adenoviral transduction resulted in the opposite finding. EA.hy926 cells infected with AdALK1-GFP show more DiI-LDL particles near the basolateral side (Fig. 7d, bottom panel). These data firmly show that ALK1 mediates LDL transcytosis.

To test if the loss of ALK1 could influence LDL uptake into the vessel wall *in vivo* we moved to genetic mouse models. Previous work has shown that genetic ablation of *Acvrl1*^fl/fl^ using an inducible global ROSA-Cre recombinase in mice leads to a rapid lethality (within 10 days) even when excised in adulthood^19^. Similarly, the breeding of *Acvrl1*^fl/fl^ mice to endothelial specific, tamoxifen inducible *Cdh5*-CreERt mice (Supplementary Fig. 8a–d) results in lethality after 10 days, as previously described^20^ precluding the ability to examine LDL uptake and atherosclerosis in adult mice. Therefore, the effects of endothelial ALK1 deletion on DiI-LDL uptake was analysed in adult *Ldlr*-KO mice after 5 days of consecutive tamoxifen injections. Under these conditions, mice appeared normal without obvious signs of haemorrhage and anaemia and BMP9 signalling was only slightly reduced in isolated EC (Supplementary Fig. 8e). Here the deletion of ALK1 in endothelium leads to significantly reduced DiI-LDL uptake into aortic endothelium as shown by *en face* confocal imaging of isolated blood vessels (Fig. 7e,f) indicating that ALK1 mediates endothelial LDL uptake *in vivo*.

## Discussion

The central goal of this study was to identify the pathways of LDL uptake by the endothelium using an unbiased, genome-wide screening approach. The rationale for identifying new pathways for LDL transit is predicated on the observations that (1) the uptake, transport and retention of sub-endothelial LDL particles can occur in an LDLR independent manner and contribute to the initiation of atherosclerosis^1^,^2^; (2) hypercholesterolemic levels of LDL will downregulate LDLR by *SREBP2*-dependent suppression of genes controlling intracellular cholesterol levels^6^ and (3) accelerated atherosclerosis is found in patients harbouring loss of function mutations in LDLR^21^ implying additional lipoprotein dependent, LDLR independent pathways of vascular disease are operational. In accordance with this hypothesis, we identified several genes that can regulate the internalization of LDL into endothelium and a majority of these genes are implicated in metabolic pathways. Detailed analysis shows that ALK1 fits the criteria for an endothelial cell specific, LDL-binding protein sufficient to promote LDL uptake and transcytosis. This is supported by knockdown or overexpression studies showing that ALK1 mediates LDL uptake and transcytosis in an LDLR and sterol sensing independent manner by the direct binding of LDL to the ectodomain of ALK1. These results define the genetic pathways that promote LDL uptake into endothelium and offers alternative strategies to modify the uptake and transport of LDL from the blood into the vessel wall.

The initial screen was designed to identify pathways that mediate rapid (60 min) LDL uptake in endothelial cells previously exposed to an excess of LDL. Using this approach, we identified multiple genes implicated in the development of atherosclerosis or in regulating cellular cholesterol levels and/or LDL uptake. Cross-referencing the genes of the follow-up list with GWAS revealed that *ARHGAP9*, *SDC1* and *SLC38A3* are linked to changes in plasma lipids, whereas *ATP6V1C1* is associated with variation in the QT-interval.

CX3CR1 is a chemokine receptor and genetic or pharmacological inhibition of this receptor attenuates atherosclerosis in mice by reducing inflammatory responses^22^,^23^,^24^. Endonuclease G is thought to be involved in DNA fragmentation during apoptosis of cells and a recent study has shown that carbamylated LDL stimulates endonuclease G (ref. 25). Leukaemia inhibiting factor upregulates hepatic LDLR in rabbits and lead to increased cholesterol clearance, resulting in decreased fatty streaks formation in the thoracic aorta^26^. Moreover, similar to findings using a genome wide, RNAi screen for genes regulating cholesterol metabolism in HeLa cells^27^, *BHMT2* and *C17orf59* were identified as genes necessary for LDL uptake in endothelial cells. About 20% (7/34) of the genes/proteins identified in the screen cluster in the vesicular trafficking pathway (highlighted in red in Supplementary Fig. 2b), indicating that these candidates are involved in intracellular transport of LDL, which could lead to another route affecting net LDL uptake in endothelial cells besides preventing the initial binding to cell membrane proteins such as LDLR or ALK1. Previous work has described a transcytotic route of LDL permeation across endothelial cells via a DMN2 dependent, caveolin-1 (CAV1)-dependent mechanism^28^,^29^. In mice deficient in CAV1, albumin and LDL uptake are reduced in isolated aortas and ^125^I-LDL and DiI-LDL uptake are reduced *in vivo*^28^,^29^ resulting in less atherosclerosis despite elevated lipids^28^,^30^. Since LDL can bind CD36 (ref. 31), the reduction in atherosclerosis in CAV1-deficient mice may be due to the reduced CD36 levels observed in *Cav1*-KO vessels^30^. Interestingly, ALK1 has been localized to caveolae and may also contribute to reduced LDL uptake in *Cav1*-knockout mice^32^. Most recently, the lipoprotein scavenger receptor for HDL, SR-B1, has been identified as a new candidate mediating the uptake and transcytosis of LDL in endothelial cells^18^. However, the loss of SR-B1 enhances atherogenesis, possibly by reducing hepatic HDL clearance^33^.

As our study focuses on the endothelium, the expression patterns of the 34 genes identified were analysed by Ingenuity Pathway Analysis and published work in MEDLINE (Supplementary Fig. 3). Only three genes are uniquely expressed in the endothelium: *ACVRL1*, *ANGPT4* and *GPR182*. ANGPT4 is the least characterized member of the angiopoietin/TIE-receptor pathway and is the human ortholog of the murine ANGPT3. Interestingly, the structure of these orthologs differ more from each other than the human and mouse counterparts of Ang1 and Ang2 (refs 34, 35, 36). This explains, why ANGPT4 has not been characterized in depth yet. Recently, GPR182 was found to be upregulated in tumour-specific endothelial cells using a microarray^37^. This GPCR has no known ligand or function in endothelial cells, but is enriched in the embryonic vasculature^38^. Future work will address the relative importance of these endothelial cell specific genes to LDL uptake and function.

Cholesterol is crucial for cell growth and maintenance, but is toxic in excess, therefore, intracellular cholesterol homeostasis is tightly regulated by the transcription factor, SREBP2. As *ACVRL1* knockdown leads to decreased internalization of LDL, the regulatory feedback on sterol sensing through SREBP2 was investigated through the expression of *SREBP2* regulated genes. Silencing of *DNM2*, effectively blocking endocytosis, leads to increased transcript levels of SREBP2-regulated genes (*LDLR*, *HMGCR*, *INSIG1* and *PCSK9*), whereas knockdown of *ACVRL1* shows no effect on sterol-mediated gene expression. Varying LDLR expression through siRNA or LDL concentration in the medium did not affect ALK1 expression or signalling, moreover the loss of ALK1 does not influence LDLR levels in extracts or the amount of LDLR on the cell surface detected by FACS. Interestingly ALK1-dependent uptake of LDL does not result in its lysosomal degradation (measured by ^125^iodotyrosine release into the media) implying the route of entry of LDL bound to ALK1 is different from LDL bound to LDLR, thus independently confirm that ALK1 does not affect sterol sensing which requires LDL degradation and metabolism of cholesterol esters. Early studies in the field suggested an LDLR independent route can account for 40–50% of LDL internalization through a non-lysosomal, non-degradative pathway^39^. This observation was corroborated by an EM-study^5^ and has been observed also in capillary endothelial cells of the blood–brain-barrier^40^.

Since ALK1 expression in *Ldlr*-KO MEFs augments LDL uptake, other components of the BMP/ALK1 pathway were examined for their effects on LDL internalization. siRNA silencing of *BMPRII*, *ActRII*, *GDF2* (BMP9)/*BMP10* or *ENG* (endoglin) in the initial screen did not affect LDL uptake implying specificity for ALK1. ALK2 was overexpressed in HeLa and HCAECs and analysed for its effect on DiI-LDL uptake and transcytosis, but the results indicate a specific effect for ALK1. In addition, ALK1-mediated LDL uptake is not affected by its kinase activity, as overexpression of constitutively active or inactive mutants and pharmacological modulation of ALK1 kinase activity had no effect on LDL uptake. The role of ALK1 signalling during atherogenesis has been suggested. For example, ALK1 expression is increased in human coronary atherosclerotic lesions^41^. However, previous studies implicating ALK1 in atherosclerosis mainly focused on signalling of the TGF-β family. Indeed, independent studies have shown that inhibition of BMP signalling attenuates the formation of atherosclerotic plaques^42^,^43^. Because ALK1 mediates LDL uptake through direct binding via its extracellular domain and transduces BMP signalling, ALK1 exerts at least two independent functions during the development of atherosclerosis. Although our data clearly demonstrates a role for ALK1 in the uptake of LDL in endothelial cells *in vitro*, examining if the loss of ALK1 influences LDL clearance and atherogenesis *in vivo* is challenging. The genetic deletion of ALK1 causes early embryonic vascular defects^44^, while conditional, post-natal deletion of ALK1 in endothelial cells also induces death within 8–10 days due to its critical role in BMP9/10 signalling^19^. Based on SPR experiments, LDL and BMP9 bind to discrete sites on LDL since the presence of BMP9 did not compete for the binding of ALK1 to LDL.

In addition to compelling *in vitro* data showing that LDL binds ALK1 and mediates LDL uptake and transcytosis, data in endothelial specific *Acvrl1*-deficient mice on a *Ldlr*-KO background demonstrates less LDL uptake into the aortic wall. Due to the lethality of sustained ALK1 inactivation in adult endothelium as seen by others^19^,^20^ and confirmed here, we could not examine if the loss of ALK1 would reduce the extent of atherosclerosis. Future studies will need to focus on separating the LDL-uptake from the BMP-signalling pathway of ALK1, which may be feasible based on the SPR data presented here.

In summary, this work defines new pathways for LDL uptake into endothelial cells and provides a molecular basis to begin unravelling LDL uptake, transcytosis and retention in the vessel wall. Although lipid lowering therapy is the mainstay for the prevention and treatment of atherosclerotic vascular disease^45^; additional therapeutic approaches targeting the early events of atherogenesis in the vessel wall such as LDL transport, retention or endothelial cell dysfunction are interesting and feasible. For example, a putative pharmacological treatment that antagonizes the LDL/ALK1 interaction in endothelial cells without affecting BMP9/BMP10-dependent signalling may afford unique and synergistic benefits with lipid lowering therapies.

## Methods

### RNAi Screen

The siGenome human genome library from Dharmacon (GE Healthcare) was used for the genome-wide RNAi screening. The images were collected with the Opera High Content Screening System (PerkinElmer) and analysed with the according Acapella software. As a primary readout the average centre intensity was determined. The primary screen was analysed based on the robust z-score^46^ per individual screening plate whereas a robust z-score≥2.5 was called a hit. Each library siRNA plate was used in three independent experiments. The quality of each plate was ensured by only accepting plates with a Z' factor>0.2, based on the control siRNA (negative control) and *DNM2* siRNA (positive control, Supplementary Table 2). The validation (with deconvoluted siRNAs) screen and the follow-up screens were analysed based on the percent effect of the positive control (*DNM2* siRNA). The secondary library siRNA plates with individual siRNAs were assed twice. To exclude the possibility of loss in signal due to low cell number cell death over 20% was not accepted and only images with at least 100 cells were taken into account.

In brief, cells (EA.hy926: 4,000 cells per well | HUVEC: 2,000 cells per well) were seeded in 384-well plates containing 20 nM siRNA (reverse transfection) with Lipofectamine RNAiMAX (Life Technologies, (1:500)). Forty-eight hours after transfection, plating the media was exchanged to a media enriched with 25 μg ml^−1^ human LDL. The concentration of 25 μg ml^−1^ was identified during the development of the high-throughput screen by using different concentrations of LDL. At 25 μg ml^−1^ the effect of LDL-pretreatment plateaued indicating that the maximum effect was reached. The identified concentration for LDLR saturation falls within the range identified by other groups^47^. Twenty-four hours later the media was removed and a serum-free media with 2.5 μg ml^−1^ DiI-LDL^48^ or 12.5 μg ml^−1^ transferrin-FITC (Tf-FITC) was added for 1 h. Cells were washed for 5 min with acid wash (25 mM Glycine, 3% (m/V) BSA in PBS at pH 4.0), fixed with 4% paraformaldehyde (PFA) for 10 min and the nuclei were stained with Hoechst for 5 min, before washed extensively with PBS before imaged. Threshold for DiI-LDL uptake was set to 50% inhibition and for Tf-FITC to 30% inhibition.

### Cells

EA.hy926 (#CRL-2922), HEK-293T (#CRL-11268) and HeLa cells (#CCL-2) were purchased from American Type Culture Collection. HUVEC were obtained from the Yale University Vascular Biology and Therapeutics (VBT) Core facility. Human coronary artery endothelial cells (HCAEC) were purchased from Lonza (#CC-2585) for ^125^I-LDL transcytosis and from PromoCell (#C-12221) for TIRF-based transcytosis. Mouse embryonic fibroblasts (MEF) from *Ldlr*-KO animals were a kind gift from Joachim Herz. MLEC were isolated^49^: Three female mice with mixed background and *Acvrl1*^fl/fl^/*Ldlr*^*−/−*^/*Cdh5*-CreERt^-^ genotype at the age of 3 weeks were euthanized using ketamine/xylazine. The lungs were isolated, minced on ice and digested using collagenase I. To isolate the CD31^+^-cells magnetic beads with anti-CD31-antibody (Supplementary Table 3) were added to the digested tissue suspension and incubated for 15 min at room temperature. By using a strong magnet, the anti-CD31-beads and CD31^+^-cells were separated and washed. The cells bound the beads were plated to allow recovery and growth. After 10 days, the cells were sorted again to achieve higher purity of CD31^+^-cells. Once the cells were confluent after the second sorting, the cells were immortalized using the polyoma middle T-antigen^13^ with polybrene for improved the transduction. Immortalized cells were selected using geneticin (0.5 mg ml^−1^).

### Generation of a stable LDLR knockdown cell line

Lentiviral vectors with five different shRNAs targeting LDLR (Supplementary Table 2) at various position of the open reading frame (ORF) were obtained from Sigma-Aldrich (MISSION). As control a scramble shRNA containing pLKO.1 vector was used. Vectors were individually cotransfected with psPAX2 (packaging) and pMD2.G (envelope) in HEK-293T cells with Lipofectamine 2,000 (Life Technologies). Virus containing supernatant was applied to EA.hy926 cells and cells were puromycin (500 ng ml^−1^) selected after 72 h.

### Animal studies

The Institutional Animal Care Use Committee of Yale University approved all mouse experiments. *Acvrl1*^fl/fl,^^50^/*Cdh5*-CreERT2 (ref. 51) animals were bred to *Ldlr*-KO animals (JAX, #007068) to generate *Acvrl1*^fl/fl^/*Ldlr*^KO/KO^/*Cdh5*-CreERT2 mice. At 8 weeks of age, littermate male mice with mixed background were injected with 100 μg g^−1^ BW tamoxifen (TMX) intraperitoneally for 5 consecutive days to induce deletion of the *Acvrl1* allele. At day 6, mice were injected via femoral vein with 100 μl of DiI-LDL (300 μg), which was allowed to circulate for 30 min. Mice were euthanized and aortae were perfused using 10 ml of PBS followed by 10 ml of 4% PFA fixation. Mouse aortae (athero-prone area; low curvature of aortic arch) were dissected and mounted *en face* following 4,6-diamidino-2-phenylindole staining. Images were acquired using confocal microscopy (Leica SP5) and quantified with the Image J. The results are expressed in terms of total positive area of each animal per field.

### Cell culture

EA.hy926 cells were cultured in DMEM (4.5 g l^−1^ glucose) supplemented with 10% fetal bovine serum (FBS), penicillin/streptomycin and glutamine (2.8 mM) and HAT-supplement (hypoxanthine, aminopterin, thymidine). EA.hy926 with lentiviral shRNA knockdown of LDLR or control shRNA (scramble) were cultured in regular EA.hy926 media with 500 ng ml^−1^ puromycin. HUVEC were cultured in M199 supplemented with endothelial cell growth supplement (Yale VBT Core Facility), 10% FBS, penicillin/streptomycin (1:100) and glutamine (2.8 mM) and used up to passage 4. For siRNA transfections HUVEC were cultured in EGM-2 media (Lonza) with 5% FBS after seeding in 384-well plates as it improves the transfection dramatically. MLEC were cultured in EGM-2 media (Lonza) with 20% FBS. HCAEC were cultured in EGM-2MV media and used in passages 5–8. HeLa cells and *Ldlr*-KO MEF were kept in DMEM (4.5 g l^−1^ glucose) supplemented with 10% FBS and penicillin/streptomycin.

### Cloning and protein expression

AdGFP and AdCre/GFP were bought from the Viral Vector Core Facility at the University of Iowa, Carver College of Medicine. Human full-length *ACVRL1* was obtained by PCR from HUVEC complementary DNA (cDNA), C terminal fused to eGFP and cloned into pENTRA1A (Invitrogen) before transferred into pAd/CMV/V5/DEST (Invitrogen) using the Gateway System (Invitrogen). To make adenovirus, HEK293T cells were transfected with Pac1-linearized adenoviral construct using Lipofectamine 2,000 (Invitrogen). After 7–10 days, the adenovirus containing supernatant was harvested, amplified by re-infection of HEK293 cells and purified with the Adeno-X Maxi Purification Kit (Clontech). The titre of the virus was determined using Adeno-X Rapid Titer Kit (Clontech) according to the instructions of the manufacturer.

ALK1-GFP was transferred from pAd/CMV/V5/DEST into pcDNA3.1 (Invitrogen) by restriction digest using BamHI/XbaI. Plasmid DNA was isolated by using ZymoPURE Plasmid Midiprep Kit (Zymo Research). Site directed mutagenesis was used to introduce various point mutations in this vector for transient expression: ALK1-Q201D (constitutively active)^44^ and ALK1-R374Q (inactive)^52^.

ALK2 (*ACVR1*) cDNA was bought from Dharmacon/GE Lifesciences (clone ID: 5209711) and fused to eGFP (pEGFP-N1, Clontech) at the carboxy-terminus by PCR and NEBuilder HiFi DNA Assembly Cloning Kit (New England Biolabs). The plasma membrane bound form of eGFP (eGFP-PM) was generated by PCR of CIBN-GFP-Stx1A^TMD^ (a kind gift of Pietro De Camilli^53^) to remove CIBN.

### siRNAs and shRNAs

All siRNAs pools used in the genome-wide RNAi screen are listed in Supplementary Data set 1. All single siRNAs used in the follow-up study are listed in Supplementary Data set 2. All other siRNAs and shRNAs are listed in Supplementary Table 2.

### Flow cytometry

The surface portion of the LDLR was measured using FACS^54^. Cells were detached from the cell culture dish using Versene (Gibco, #15040066), before the cells were labelled with anti-LDLR antibody or mouse IgG2b as a control (Supplementary Table 3). After incubation for 1 h, the cells were washed to remove unbound antibodies, before the secondary antibody (Supplementary Table 3) was added and incubated for additional 30 min. The cells were washed again, resuspended in PBS and analysed immediately. If DiI-LDL uptake into cells was determined by FACS, cells were washed with PBS, treated with DiI-LDL (2.5 μg ml^−1^) for 1 h, washed with acid wash (25 mM Glycine, 3% (m/V) BSA in PBS at pH 4.0), washed with PBS, before trypsinized, and resuspended in PBS before analysed immediately. Cells were measured by FACS Calibur or LSRII (both BD Bioscience) flow cytometer and analysed using FlowJo software.

### Surface plasmon resonance

Binding studies were performed at 25 °C using a Biacore T100 optical biosensor (GE Healthcare) equipped with a CM5 research-grade sensor chip coated with LDL. The LDL surface was created using amine coupling and equilibrated with running buffer (25 mM HEPES, pH 7.4, 150 mM NaCl and 0.1 mM CaCl_2_) (ref. 17). The binding of LDLR^ecto^ or ALK1^ecto^ was monitored during injections of five different concentrations each (Fc: 0.1, 0.2, 0.6, 1.8, 5.4 μM; ALK1^ecto^: 0.19, 0.57, 1.7, 5.1, 15.3 μM; LDLR^ecto^: 5, 14, 43, 129, 388 nM) using single cycle kinetics (no regeneration steps). The binding responses were double-referenced against the non-specific binding to dextran surface alone and injections of buffer alone. The resulting sensorgrams were fit to a simple 1:1 binding model to determine apparent binding affinity using BioEvaluation software (GE Healthcare). To establish the non-competitive nature of ALK1^ecto^ and LDLR^ecto^ binding to LDL, the binding of ALK1^ecto^ was monitored on native LDL or on LDL that was saturated with LDLR^ecto^ before ALK1^ecto^ binding experiments and vice versa. The binding of Fc, ALK1^ecto^, BMP9 and ALK1^ecto^ pre-incubated with equimolar amounts of BMP9 were tested at 2 μM protein concentration in the presence of 0.48 mM hydrochloric acid (needed to reconstitute the lyophilized BMP9). Due to the complex nature of buffers used in preparation of lyophilized proteins used in this experiment that prevented proper referencing of the association signal, the binding of Fc, ALK1^ecto^ and BMP9 were quantitated at the stability reference point (initial phase of the dissociation step) to eliminate the responses due to bulk refractive index change due to buffer differences between protein samples and the running buffer; these responses were normalized against the amplitude generated during ALK1^ecto^ binding.

### Experimental procedure using ^125^I-LDL

Native LDL was bought from Kalen Biomedical and labelled with 125-iodine by PerkinElmer as a customized order. The activity at the time of delivery was about 3 μCi μg^−1^ protein. Uptake, binding and degradation were measured as described in a detailed protocol published before^55^.

*Uptake studies*. Cells were washed with PBS and treated with plain DMEM containing 5 μg ml^−1 125^I-LDL for 1 h at 37 °C. Cells were acid washed (25 mM Glycine, 3% (m/V) BSA in PBS at pH 4.0), washed with PBS and lysed with 0.1 M NaOH. A part of the lysate was used to determine the protein concentrations (Bio-Rad Protein Assay). The rest of the lysate was mixed with Ultima Gold scintillation liquid (PerkinElmer) before measured using a Tri-Carb 2100 liquid scintillation counter (PerkinElmer).

*Binding studies*. Cells were washed with PBS and treated with plain DMEM containing 0–100 μg ml^−1^ LDL with a fixed amount of ^125^I-LDL (‘spiked') for 3 h at 4 °C. After the incubation, cells were washed five times with buffer A (150 mM NaCl, 50 mM Tris-HCl, 2 mg ml^−1^ BSA, pH 7.4) and once with buffer B (150 mM NaCl, 50 mM Tris-HCl, pH 7.4). Cells were lysed as described before, protein concentration was measured and lysate was counted.

*Degradation studies*. Cells were washed with PBS and treated with plain DMEM containing 5 μg ml^−1 125^I-LDL for 4 h at 37 °C. The media was collected in a glass tube, LDL particles were precipitated with trichloroacetic acid and centrifugation. Remaining supernatant contained free ^125^I from the initial synthesis or due to degradation and ^125^I-tyrosine. ^125^I-tyrosine can be used as readout for degradation as it cannot be recognized by transfer RNA and will, therefore, leak into the media^56^. Free ^125^I^-^ is removed by oxidizing it to I_2_ in an excess of KI (I^-^) with H_2_O_2_. I_2_ is separated from I^-^ by adding chloroform. The lower phase will turn purple and contains the I_2_. The aqueous phase (top) should be colourless or light yellow and contains the ^125^I-tyrosine, which can be measured as described above. The remaining cells are lysed and the protein concentration is measured to normalize the measurements.

### Transcytosis of ^125^I-LDL

Native LDL was bought from Kalen Biomedical and labelled with 125-iodine from PerkinElmer using chloramine-T^57^. The ^125^I-LDL was only used, if >98% of the radioactivity could be precipitated by trichloroacetic acid and the counts were between 200-600 c.p.m. ng^−1^ (= 90–270 pCi ng^−1^) (ref. 55). HCAECs were seeded ad a density of 20,000 cells per insert onto 0.4 μm pore polyester transwells (0.33 cm^2^) and grown until confluency (0.1 ml upper chamber/0.6 ml lower chamber), before ^125^I-LDL (25 μg ml^−1^) was added and cells were incubated at 37 °C for 5 h. Cells were kept in LPDS+25 μg ml^−1^ LDL overnight before the experiment. LDL transcytosis was determined by measuring the radioactivity of 100 μl in the lower chamber using a scintillation counter. Paracellular leakage was assessed by adding 70 kDa FITC-labelled dextran to each insert (upper chamber) at a concentration of 50 μg ml^−1^. An aliquot of 100 μl from the lower chamber was measured using a fluorescent plate reader (ex: 485/20 nm and em: 528/20 nm). Only samples with FITC-dextran concentrations below 0.2 μg ml^−1^ were used for transcytosis assays to rule out any participation of paracellular permeability in LDL flux.

### TIRF-based transcytosis of DiI-LDL

For TIRF imaging experiments, HCAECs were seeded on 18 mm glass coverslips and used for imaging during passages 5–8. HEK293 cells were grown on 100 mm dishes and maintained in standard low-glucose DMEM with serum. For some experiments, LDL was isolated from freshly drawn plasma from healthy adult volunteers using a procedure described by Vieira *et al*.^58^; written informed consent was obtained and the study was approved by the institutional Research Ethics Board (St Michael's Hospital REB#14-278). Briefly, plasma density was adjusted to 1.21 g ml^−1^ by adding solid KBr after adding 1 mM EDTA into plasma in order to prevent oxidation. The plasma solution was then distributed into 10.4-ml polycarbonate centrifuge tubes and a discontinuous density gradient was made by overlaying the plasma solution (4 ml) with 6.4 ml of saline-EDTA containing 110 mM NaCl, 20 mM phosphate, pH 7.4 and 0.3 mM EDTA. The tubes were ultracentrifuged in a Beckman L-80 ultracentrifuge equipped with a 70.1 Ti fixed angle rotor at 65,000 r.p.m. for 3 h at 15 °C. The isolated LDL was concentrated by ultrafiltration and dialysed against saline-EDTA, and then filtered with a 0.45 μm syringe filter. LPDS was then prepared from human plasma by ultracentrifugation and dialysed against saline-EDTA buffer, and similarly filtered. LDL was labelled with DiI (ab145311, Abcam) using a modified procedure described by Pitas *et al*.^59^; 1 mg of LDL was added to 2 ml of LPDS and 50 μl of DiI (3 mg ml^−1^ in dimethylsulphoxide) was added while the solution was gently vortex-mixed. After incubating this mixture in the dark for 18 h at 37 °C, the DiI-labelled LDL (DiI-LDL) was isolated by ultracentrifugation (35,000 r.p.m., 24 h, 4 °C. Beckman SW55 Ti rotor), dialysed against saline-EDTA, and filter-sterilized. The protein concentration of LDL and DiI-LDL was determined by the BCA (bicinchoninic acid assay) method; the homogeneity of LDL and DiI-LDL was determined via agarose gel electrophoresis. Fluorescence of DiI-LDL diluted in 0.1% SDS in 0.1 M NaOH was measured with excitation and emission wavelengths at 520 and 580 nm, respectively^60^. The incorporation of DiI into LDL was 35 ng DiI μg^−1^ LDL-protein. LDL and DiI-LDL were stored at 4 °C in the dark and used within 2 months. These preparations were indistinguishable functionally from commercially obtained LDL and DiI-LDL but were of more consistent quality (for example, higher concentration, brightness); they were, therefore, preferentially used for TIRF experiments.

For siRNA experiments, HCAECs at 60% confluency were transfected in Opti-MEM with 16 nM *ACVRL1* siRNA or scrambled control siRNA using HiPerFect. LDL transcytosis was measured by TIRF imaging 72 h after transfection. For overexpression experiments, HCAECs at 80% confluency were transfected in Opti-MEM with 0.5 ug ml^−1^ plasmid DNA (GFP, ALK1-GFP, ALK2-GFP) using a 6:1 ratio of HiPerFect to DNA. LDL transcytosis was measured by TIRF imaging 24 h after transfection. To prepare PCSK9 conditioned media, HEK293 cells were transfected in serum free DMEM with 1 μg ml^−1^ PCSK9 plasmid (a gift from N. Seidah, IRCM) using X-tremeGENE HP. After 4 h the transfection media was aspirated and 20 ml fresh DMEM with serum was added. PCSK9 conditioned DMEM was collected 24 h after transfection and frozen in single-use aliquots.

LDL transcytosis by confluent primary human coronary artery endothelial cells was measured by total internal reflection fluorescence (TIRF) microscopy^18^,^61^. Briefly, confluent HCAECs seeded on 18 mm coverslips were placed in a cell chamber and treated with 40 μg ml^−1^ DiI-LDL and 5 μl NucBlue in cold RPMI 1640 media with HEPES at 4 °C for 10 min to allow the DiI-LDL to bind to the apical cell membrane without internalization. Cells were rinsed three times with PBS to wash away unbound DiI-LDL followed by the addition of 500 μl warm RPMI. The chamber was placed on a 37 °C live cell imaging stage for 2 min before imaging. TIRF images were acquired on an Olympus Cell TIRF Motorized module mounted on an Olympus IX81 microscope using a 150 × objective and 561 nm excitation laser and a penetration depth of ∼110 nm. In siRNA experiments, the 4,6-diamidino-2-phenylindole epifluorescent channel was used to randomly select cells for imaging; 15 TIRF videos were taken per condition. In overexpression experiments, transfected cells were identified using the green epifluorescent channel; 10-12 TIRF videos were taken per condition.

Quantification of LDL transcytosis was performed and has been previously described in detail^1^,^2^. Briefly, a MATLAB algorithm (written by Bryan Heit, Western University, Canada) was used to track individual LDL-containing vesicles as they enter the TIRF field. Vesicles are filtered based on size, circularity and fluorescent intensity above background; vesicles undergoing fusion with the basal membrane are identified by a sudden decrease in fluorescent intensity over two consecutive frames, greater than 2.5 s.d. than the rate of fluorescence decrease of the vesicle over the entire period that the vesicle has been tracked. Confounding by endocytic traffic is excluded by requiring vesicles to be stationary before fusion (that is, docking with the membrane). In control HCAECs, 20–40 exocytosis events are typically captured in 150 frames taken at 6.67 frames per second.

Cell lysates were collected from coverslips immediately following TIRF imaging. Samples from every two experiments were pooled. SDS–polyacrylamide gel electrophoresis was performed on an 8% polyacrylamide gel and transferred onto a nitrocellulose membrane. The membrane was blocked with 5% BSA for 1 h at room temperature, followed by overnight incubation at 4 °C with anti-human LDLR antibody or anti-β-actin antibody (Supplementary Table 3). Membranes were washed with TBST followed by incubation with HRP-conjugated secondary antibodies for 1 h at room temperature. After several washes in TBST, membranes were visualized with enhanced chemiluminescence detection.

### Cross-section imaging

Cross-section imaging (*x*/*y*-axes) was performed following previous published work^18^ with a few modifications. In brief, EA.hy926 were either transfected with control siRNA or *ACVRL1* siRNA or infected with adenovirus encoding GFP or ALK1-GFP on coverslips. Cells were starved in serum-free media for 4 h before they were incubated with DiI-LDL (2 μg ml^−1^) at 4 °C for 10 min to allow membrane binding. Cells were then treated with serum-free media and placed in the incubator at 37 °C for 30 min, then washed three times with PBS, fixed and stained with 20 μg ml^−1^ lectin (Vector Labs, UEA I (ref. 62)). Cells were stained with counterstained with Hoechst, mounted on slides, and imaged using an SP5 confocal microscope. Images were acquired with a z-stack interval of 0.3 μm. Image sets were reconstructed using Velocity and *x*/*y* cross-sections were extracted.

### Genotyping

*Acvrl1* floxed alleles, *Ldlr*-KO allele and *Cdh5*-CreERT2 were genotyped by PCR using primers and the PCR programs described in Supplementary Table 4.

### Quantitative PCR analyses

Cells were washed with PBS, messenger RNA was isolated with RNeasy Mini Kit (Qiagen), cDNA was reverse transcribed with iScript cDNA Synthesis Kit (Bio-Rad) before used with IQ SYBR Green Supermix (Bio-Rad) and the quantitative PCR primers listed in Supplementary Table 5 on an iQ5 cycler (Bio-Rad).

### Immunoblotting

For immunoblotting, tissues or cells were placed in lysis buffer (50 mM Tris-HCl, 1% NP-40, 0.1% SDS, 0.1% Na-deoxycholate, 0.1 mM EDTA, 0.1 mM EGTA, 200 μM NaF, 20 μM Na-pyrophosphate, 2 mg ml^−1^ complete protease inhibitor, 0.3 mg ml^−1^ Pefabloc phosphatase inhibitor, 40 mM β-glycerophophate, 2 mM Na_3_VO_4_). Tissues were homogenized before sonication. Cell lysates were centrifuged for 15 min at top-speed at 4 °C. Protein concentrations were determined (Bio-Rad Protein Assay). Equal amounts of protein were separated by 10% SDS–polyacrylamide gel electrophoresis or 4-20% gradient gels (Bio-Rad) and transferred to nitrocellulose membranes. After Ponceau S staining, the membranes were blocked with 1% casein for 1 h at room temperature under continuous rotation before incubation with the primary antibody O/N at 4 °C. Supplementary Table 5 lists all primary antibodies used in this study. LI-COR compatible secondary antibodies (680 or 800 nm) were used based on species origin of the primary antibody. All antibodies in this study are listed in Supplementary Table 3.

### Immunofluorescence

Cells were washed with PBS, fixed with PFA (4%) for 10 min, permeabilized with 0.2% Triton-X-100 before stained with antibodies listed in Supplementary Table 5 using a regular immunofluorescence protocol and imaged with a confocal laser scanning microscopy (CLSM). Respective secondary antibodies were applied before Hoechst staining and subsequent imaging, as previously described. If quantitative assessments were performed, CellProfiler^63^,^64^ was used to determine the intensity of blue (Hoechst), green (GFP), red (DiI) for further analyses. Pearson correlation was also analysed using CellProfiler. Both CellProfiler pipelines can be requested. All antibodies in this study are listed in Supplementary Table 3.

### Filipin-III Staining

Filipin-III was used to visualize free cholesterol in cells^65^. A 25 mg ml^−1^ stock solution of Filipin-III in dimethylsulphoxide was prepared and kept at −20 °C. Cells were washed twice with PBS, fixed with PFA (4%) for 10 min, washed twice with PBS, permeabilized with 0.1% Triton-X-100 in PBS for 5 min at room temperature, washed twice with PBS, before stained with 50 μg ml^−1^ (1: 500) Filipin-III for 1 h in the dark at room temperature. Cells were washed twice with PBS, before imaged immediately (staining fades rapidly) with epifluorescence microscope.

### Chemicals

All chemicals, in the highest grade of purity, were obtained from Sigma-Aldrich unless otherwise noted. The human LDL, DiI-LDL, DiI-OxLDL, DiI-VLDL, DiI-HDL and LPDS were bought from Kalen Biomedical. Fc-fragment, ALK1^ecto^-Fc, BMP9, and LDLR^ecto^-(His)_6_ were obtained from R&D Systems. LDN193189 was bought from Selleckchem.

### Statistics

The results are expressed as means±s.e.m. of at least three independent experiments. Statistical analyses were performed with Prism 6 software (GraphPad) using the two-tailed, unpaired Student's *t*-test. *P* values<0.05 were considered statistically significant and marked with an asterisk (*).

### Data availability

The original data sets used for the initial genome-wide screen and the follow-up screen can be found in the supplementary information. All images of the high-throughput screen are stored on a Yale server, which is backed up regularly to ensure long term accessibility. All relevant data are available from the authors upon request.

## Additional information

**How to cite this article:** Kraehling, J. R. *et al*. Genome-wide RNAi screen reveals ALK1 mediates LDL uptake and transcytosis in endothelial cells. *Nat. Commun.* **7,** 13516 doi: 10.1038/ncomms13516 (2016).

**Publisher's note:** Springer Nature remains neutral with regard to jurisdictional claims in published maps and institutional affiliations.

## Supplementary Material

Supplementary Information Supplementary Figure 1-12, Supplementary Table 1-5, and Supplementary References

Supplementary Movie 1 TIRF videos of LDL transcytosis in HCAECs transfected with control siRNA. DiI-LDL was added to the apical surface of the cell monolayer as described in the Methods. This video shows vesicles containing DiI-LDL entering the basal 110 nm of the cell. Here they may fuse with the basolateral membrane, thus completing transcytosis, or undergo free Brownian diffusion either remaining in the field of view or leaving it. The video contains 150 images taken at a frame rate of 6.67/second.

Supplementary Movie 2 TIRF videos of LDL transcytosis in HCAECs transfected with ALK1 siRNA. DiI-LDL was added to the apical surface of the cell monolayer as described in the Methods. This video shows vesicles containing DiI-LDL entering the basal 110 nm of the cell. Here they may fuse with the basolateral membrane, thus completing transcytosis, or undergo free Brownian diffusion either remaining in the field of view or leaving it. The video contains 150 images taken at a frame rate of 6.67/second.

Supplementary Data 1 The excel spreadsheet shows the original data from the three independent repeats of the genome-wide screen. The data are presented as robust z-score [RZ] as described in the method section. The plate ID and well are unique identifiers for the images used to calculate the RZ.

Supplementary Data 2 The excel spreadsheet shows the data from the follow-up screen performed in duplicates (Set 1 and Set 2) with individual siRNAs. The data are presented in percent inhibition. Gene candidates were checked in EA.hy926 cells by using individual siRNAs (red column). Only genes downregulated by at least 2 siRNAs showing an effect on DiI-LDL uptake equal or greater than 50% in both replicates were used for further analysis (highlighted in yellow). Gene candidates were checked in EA.hy926 cells for their effect on transferrin uptake by using individual siRNAs (green column). Only genes downregulated by at least 2 siRNAs showing an effect on transferrin-FITC uptake less than 30% in both replicates were used for further analysis (highlighted in yellow). Gene candidates were checked in HUVECs on their effect on DiI-LDL uptake by using individual siRNAs (orange column). Only genes downregulated by at least 2 siRNAs showing an effect on DiI-LDL uptake equal or more than 50% in both replicates were used for further analysis (highlighted in yellow). The blue column represents the data from EA.hy926 with either high expression of LDLR or low expression of LDLR. If the knockdown of at least 2 siRNAs resulted in a 2-fold difference between the conditions of high- or low LDLR expressing cells, these genes were considered to be LDLR dependent and excluded from further analysis (highlighted in red).

Supplementary Data 3 The excel spreadsheet shows the analysis of publically available GWAS data for genes identified in this study. References are given in the 2nd column.
