# Supplementary material for: Genome-wide RNAi screen reveals ALK1 mediates LDL uptake and transcytosis in endothelial cells
Source: Nat Commun. 2016 Nov 21;7:13516. doi: 10.1038/ncomms13516 (PMC5121336; doi:10.1038/ncomms13516)
Supplement: Supplementary Information — Supplementary Figure 1-12, Supplementary Table 1-5, and Supplementary References [file ncomms13516-s1.pdf]

Supplementary Figure 1

a

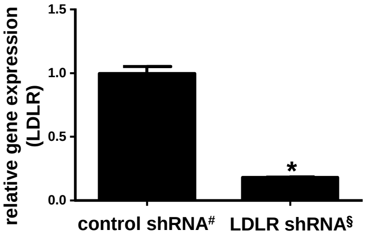

b

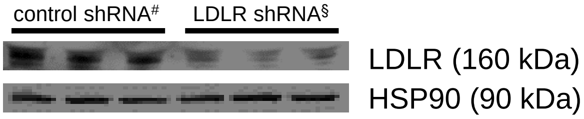

Supplementary Fig. 1. EA.hy926 cell line with LDLR knockdown.
a) qPCR result for EA.hy926 transduced with either scramble shRNA lentivirus or the 5 shRNA lentiviruses targeting the ORF of LDLR. #, cells cultured in LPDS. §, cells cultured in LDL supplemented media (25 µg/ml). Data represent the mean ± SEM and are representative of 3 experiments in duplicate. *$p < 0.05$, Student's t-test. b) Western blot analysis for EA.hy926 transduced with either scramble shRNA lentivirus or the 5 shRNA lentiviruses targeting the ORF of LDLR. #, cells cultured in LPDS. §, cells cultured in LDL-supplemented media (25 µg/ml) A non-cropped western blot for this experiment can be found in Supplementary Figure 11b.

Supplementary Figure 2

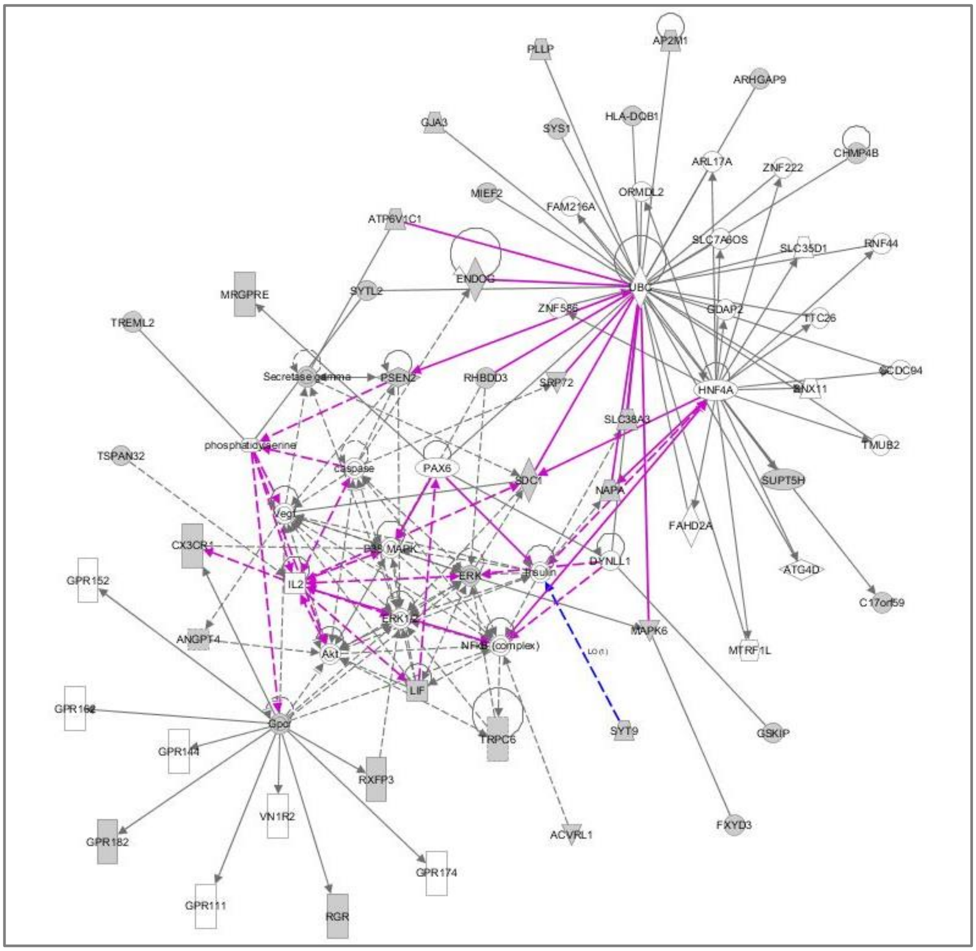

Supplementary Fig. 2. Qiagen's Ingenuity Pathway Analysis.
Analysis of the 34 gene hits revealed 3 distinct networks. Representation in a gene cluster format.

Supplementary Figure 3

Gene symbol	E	T	H	L	S	CVD	Lipids
ABCC8	1	2	1	0			
ABCG1	0	2	1	0			
ABCG5	2	2	1	0		CAD	LDL, TC
ABHD12B	1	2	2	0			
ACVRL1	4	1	4	0	1		
ADORA1	1	4	2	0			
ANGPT4	2	1	4	0	2		
ANTXR2	2	4	1	0		SBP, DBP, MAP	
AP2M1	2	0	4	0	3		
APOB	0	2	0	0		CAD	LDL, HDL, TG, TC
ARCN1	4	4	4	0			TC
ARHGAP35	0	4	1	0			
ARHGAP9	2	1	4	0	4		HDL, TG
ATP6V1C1	2	1	4	0	5	QT	
BHMT2	2	4	4	0			
BTN3A1	2	3	3	0			
C12orf52	0	0	3	0			
GSKIP (C14orf129)	2	1	2	0	6		
C15orf38	2	2	3	0			
C17orf59	3	1	3	0	7		
C17orf80	1	4	2	1			
C19orf6 (TMEM259)	1	3	4	0			
C21orf2	2	2	3	0			
C9orf125 (TMEM246)	2	2	3	0			
CAV1	0	3	0	0		A-fib, PR & QT	TG
CAV2	1	4	2	3			
CAV3	1	2	1	0			
CCDC30	0	2	1	0			
CDC42BPB	0	0	0	0			
CDH23	0	3	1	0			
CHMP2A	2	3	2	0			
CHMP4B	4	0	4	0	8		
CHMP5	1	0	0	0			
CHRNA9	0	3	2	0			
CLEC6A	1	3	3	0			

Gene symbol	E	T	H	L	S	CVD	Lipids
CLIC1	1	2	2	0			
CLMN	1	1	0	1			
COG1	1	3	2	0			
CRELD1	0	3	1	2			
CSNK1E	1	0	2	0			
CX3CR1	2	1	3	0	9		
CXorf66	0	1	1	1			
DOK6	0	1	0	1			
DPP4	0	1	1	0			
DPYSL2	1	2	4	0			
DSTYK	0	3	0	1			
DUSP19	1	0	1	0			
EGFL6	1	0	2	0			
EGFR	0	3	2	0			
ELK1	2	2	0	1			
ENDOG	3	0	2	0	10		
FAM18B1	0	1	2	0			
FCRL1	1	0	0	0			
FCRL3	1	3	0	1			
FXYD3	3	0	3	0	11		
GJA3	4	0	3	0	12		
GPR182	2	1	2	0	13		
GSK3B	0	2	0	0			HDL
HIP1	2	1	1	0			
HLA-DQB1	3	1	2	0	14		
INADL	0	4	1	0			
IRF6	1	0	0	0			
IRF7	0	1	0	0			
ITGB5	2	2	2	0			
ITM2C	2	3	3	0			
KBTBD3	0	0	0	1			
KLRB1	0	0	0	0			
LEPROT	3	3	1	0			
LIF	3	0	4	0	15		
LPHN3	0	0	2	1			

Gene symbol	E	T	H	L	S	CVD	Lipids
LPL	1	2	4	1		CAD, PP	TG, HDL
MAP2K1	3	2	2	2			
MAPK6	2	1	2	0	16		
MAPK8	1	0	4	0			
MARK4	3	2	2	0			
MRGPRE	2	0	4	0	17		
MUSK	1	4	2	1			
NAPA	4	0	4	0	18		
NID1	0	1	1	0			
NOTCH4	0	0	4	0			
NPFFR2	0	2	0	1			
NTN3	1	2	4	0			
NYX	1	2	4	1			
OR52N4	2	2	1	0			
OR5C1	0	0	1	0			
PALLD	0	2	0	0			
PDIA3	1	0	2	0			
PISD	1	1	2	0			
PLD6	0	1	0	1			
PLLP	2	1	3	0	19		
PPIAL4A	1	1	0	0			
PQLC3	3	2	2	0			
PRKCDBP	1	1	1	0			
PSAP	0	1	2	0			
PSEN2	2	1	3	0	20		
PTRF	0	2	3	0			
RAB7A	1	1	1	0			
RASGEF1B	0	1	0	1			
RELA	1	2	2	0			HDL
RGR	2	0	4	0	21		
RHBDD3	4	0	4	0	22		
RXFP3	2	0	3	0	23		
SARM1	1	1	4	0			
SCARB1	1	2	3	1			HDL
SCLT1	0	1	0	0			

Gene symbol	E	T	H	L	S	CVD	Lipids
SDC1	2	1	3	0	24		TC
SDPR	1	3	1	0			
SEMA5A	1	0	2	0			
SERINC2	1	0	2	0			
SFN	0	1	0	0			HDL, LDL, TG
SHANK1	0	2	0	0			
SHANK3	1	0	1	1			
SHROOM4	0	1	0	0			
SLC10A1	1	0	2	1			
SLC10A2	1	0	3	0			
SLC10A5	0	2	2	0			
SLC38A3	2	0	4	0	25		HDL
MIEF2 (SMCR7)	3	1	4	0	26		
SMR3B	1	1	3	0			
SRP72	2	0	3	0	27		
SSTR4	2	2	3	2			
STAT3	1	1	3	0			
SUPT5H	4	0	4	0	28		
SYS1	3	0	3	1	29		
SYT9	3	0	3	0	30		
SYTL2	3	1	2	0	31		
TMEM179B	0	2	1	0			
TMEM18	0	2	1	0			
TMEM184C	0	0	1	0			
TMEM89	2	2	3	0			
TRAPPC4	1	0	0	1			
TREML2	3	0	2	0	32		
TRPC6	2	0	3	0	33		
TSPAN12	1	1	0	0			
TSPAN32	2	1	3	0	34		
TSPEAR	1	1	4	0			
UFSP2	0	2	2	0			
VPS29	0	1	2	0			
VPS39	1	1	1	1			
WDR26	4	2	4	0			

E: DiI-LDL uptake in EA.hy926 cells
T: Transferrin-FITC uptake in EA.hy926 cells
H: DiI-LDL uptake in HUVECs
L: LDLR dependency
S: endothelial specific

**CVD**	cardiovascular traits
**Lipids**	effect on lipids
**A-fib.**	atrial fibilliation
**CAD**	coronary artery disease
**DBP**	diastoloic blood pressure
**HDL**	high-density lipoprotein
**LDL**	low-density lipoprotein
**MAP**	mean arterial pressure
**PP**	pulse pressure
**PR**	PR interval
**QT**	QT interval
**SBP**	systolic blood pressure
**TC**	total cholesterol
**TG**	triglycerid

Shown are the numbers of individual siRNAs targeting each gene that score positive in each screen. Green, siRNAs fullfil the assay criteria (**E** + **H**: ≥ 2 positive siRNAs / **T** + **L**: ≤ 2 positive siRNAs). Red, siRNAs does not fullfil the assay criteria (**E** + **H**: ≤ 2 positive siRNAs / **T** + **L**: ≥ 2 positive siRNAs).

Supplementary Fig. 3. Follow-up screen.
140 genes from the initial GW RNAi screen were further analysed. See Supplementary Datatset 2 for further details. References for the expression pattern are listed under Supplementary References at the end of this document. See Supplementary Dataset 3 for further details on the GWAS data.

Supplementary Figure 4

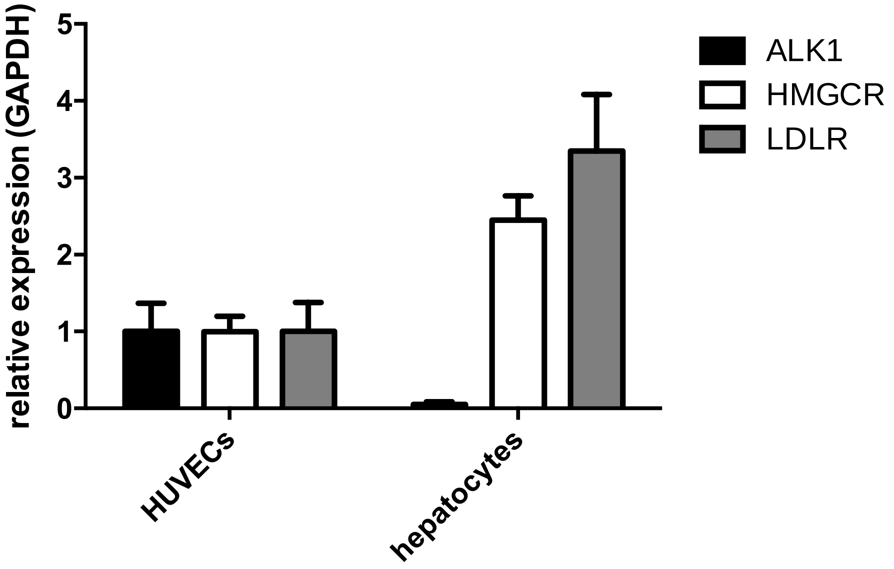

Supplementary Fig. 4. Transcript levels for ALK1 in human primary endothelial cells and hepatocytes
The transcript levels of *ACVRL1*, *HMGCR* and *LDLR* were compared between primary human endothelial cells (HUVEC) and primary human hepatocytes (healthy donors).

Supplementary Figure 5

a

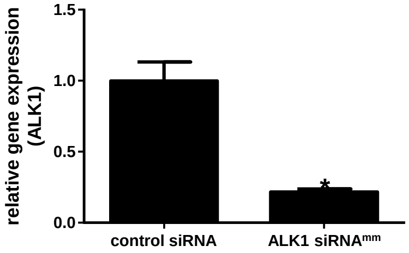

b

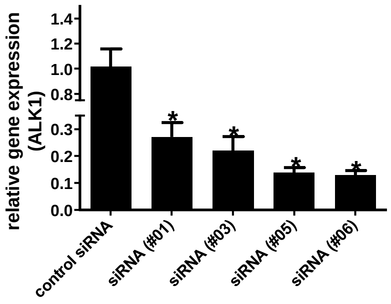

c

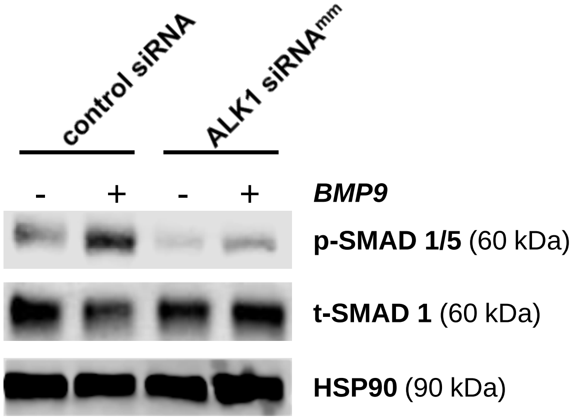

Supplementary Fig. 5. siRNA efficiency analysis

a) qPCR analysis of the knockdown efficiency of siRNA against murine ALK1 (here MLEC). Data represent the mean ± SEM and are representative of 3 experiments in duplicate. *$p < 0.05$, Student's t-test. b) qPCR analysis of all 4 individual siRNAs against human ALK1 used in the GW RNAi screen. Analysis performed in HUVECs. Data represent the mean ± SEM and are representative of 3 experiments in duplicate. *$p < 0.05$, Student's t-test. c) Western blot analysis of the knockdown efficiency of siRNA against murine ALK1 (here MLEC) based on the BMP9 (10 ng/ml) induced phosphorylation of SMAD 1/5. A non-cropped western blot for this experiment can be found in Supplementary Figure 12a.

Supplementary Figure 6

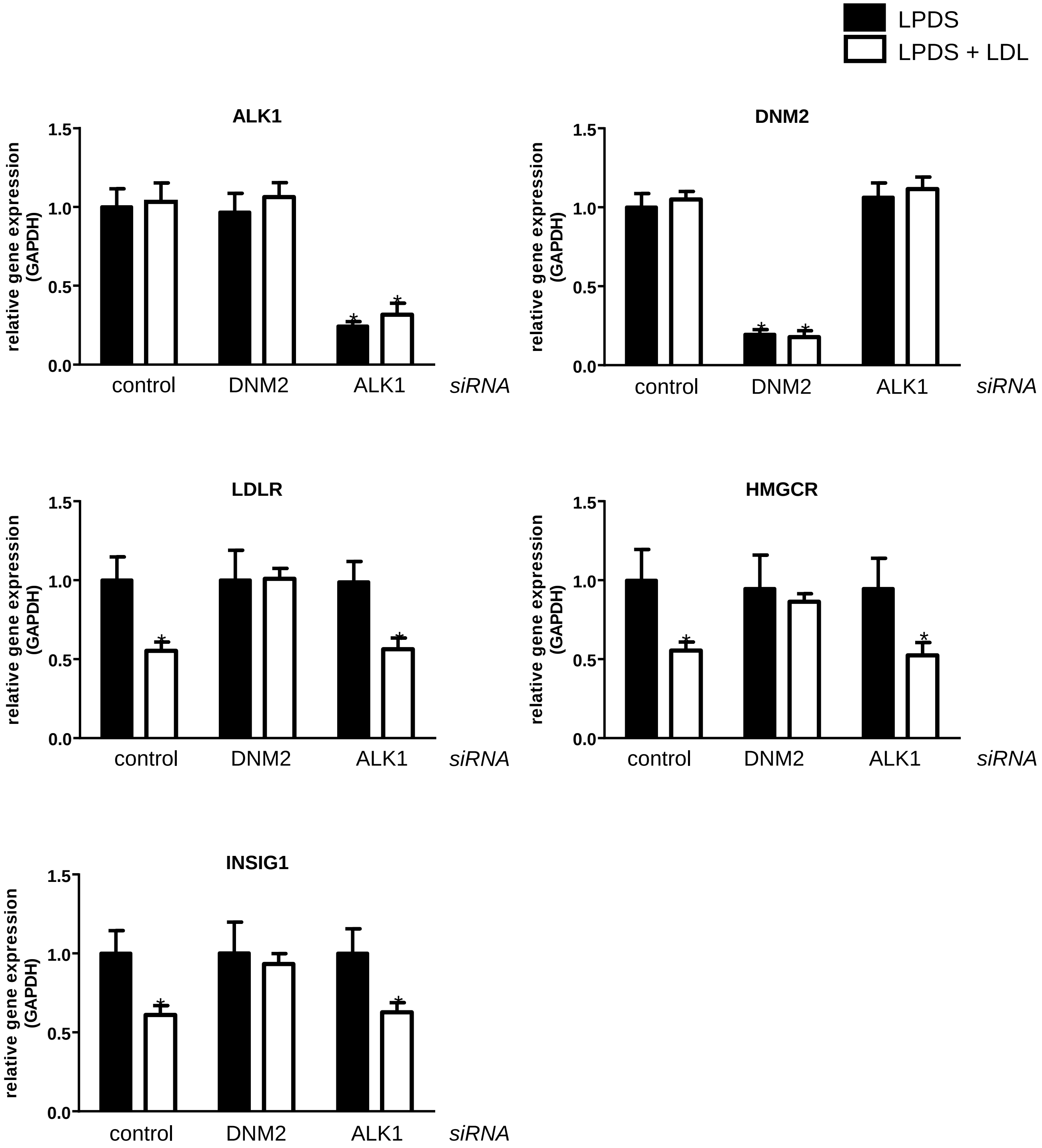

Supplementary Fig. 6. ALK1 knockdown does not affect transcript levels of SREB2-dependent genes Cells were treated with control siRNA, *ACVRL1* siRNA and *DNM2* siRNA and kept in either low LDL media (LPDS) or high LDL media (LDL pretreated), before transcripts for genes involved in sterol sensing were analysed. Data represent the mean ± SEM and are representative of 3 experiments in duplicate. *$p < 0.05$, Student's t-test.

Supplementary Figure 7

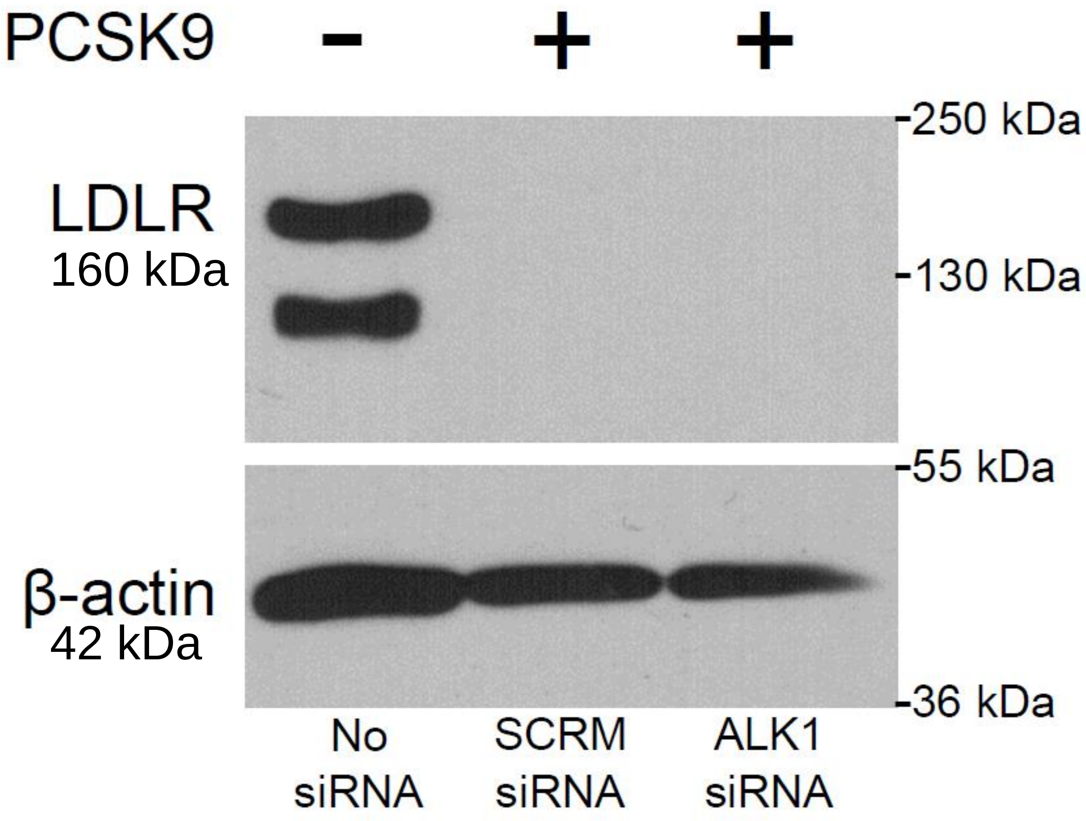

Supplementary Fig. 7. PCSK9 conditioned media leads to degradation of LDLR in HCAECs
Treatment of HCAECs transfected with control siRNA or *ACVRL1* siRNA with PCSK9 conditioned media for at least 6 hrs results in a complete removal of LDLR.

Supplementary Figure 8

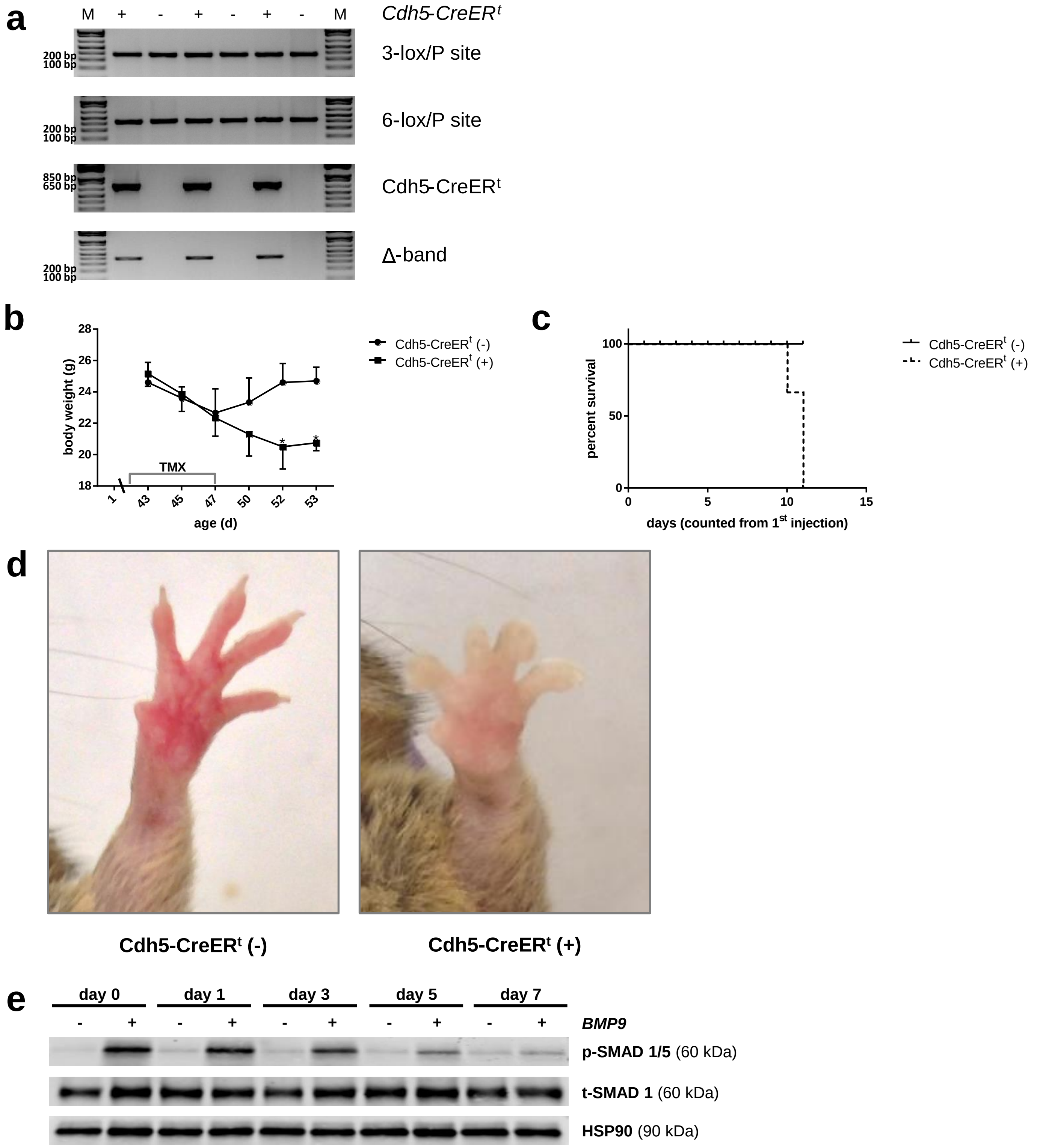

Supplementary Fig. 8. Endothelial specific deletion of ALK1 in adult mice leads to rapid death
a) Genotyping results of *Acvrl1*$^{fl/fl}$ mice with or without the inducible *Cdh5*-CreERT2 transgene 7 days after first TMX injection. b) Weight measurements. Data represent the mean ± SEM and are representative of 3 animals each. *$p < 0.05$, Student's t-test. c) Kaplan-Meier curve. d) Representative images of paws from Cre-negative and Cre-positive animals 7 days after the first TMX injection. e) Cre-positive animals were injected with TMX for 0, 1, 3, 5 or 7 days and primary MLECs were isolated and treated after 3 hrs of starvation with vehicle or BMP9 (10 ng/ml). A non-cropped western blot for this experiment can be found in Supplementary Figure 12b.

Supplementary Figure 9

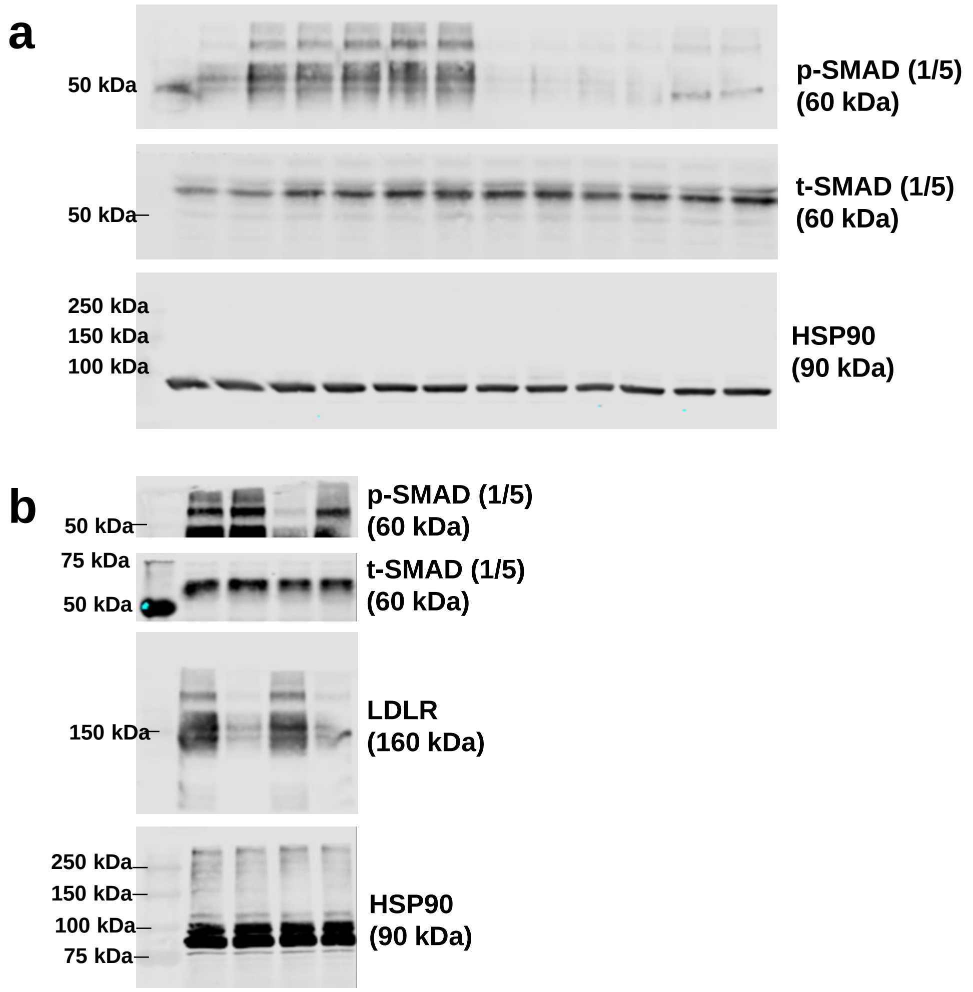

Supplementary Fig. 9. Original Western Blots

a) Original gel scans for Fig. 2b. b) Original gel scans for Fig. 3b.

Supplementary Figure 10

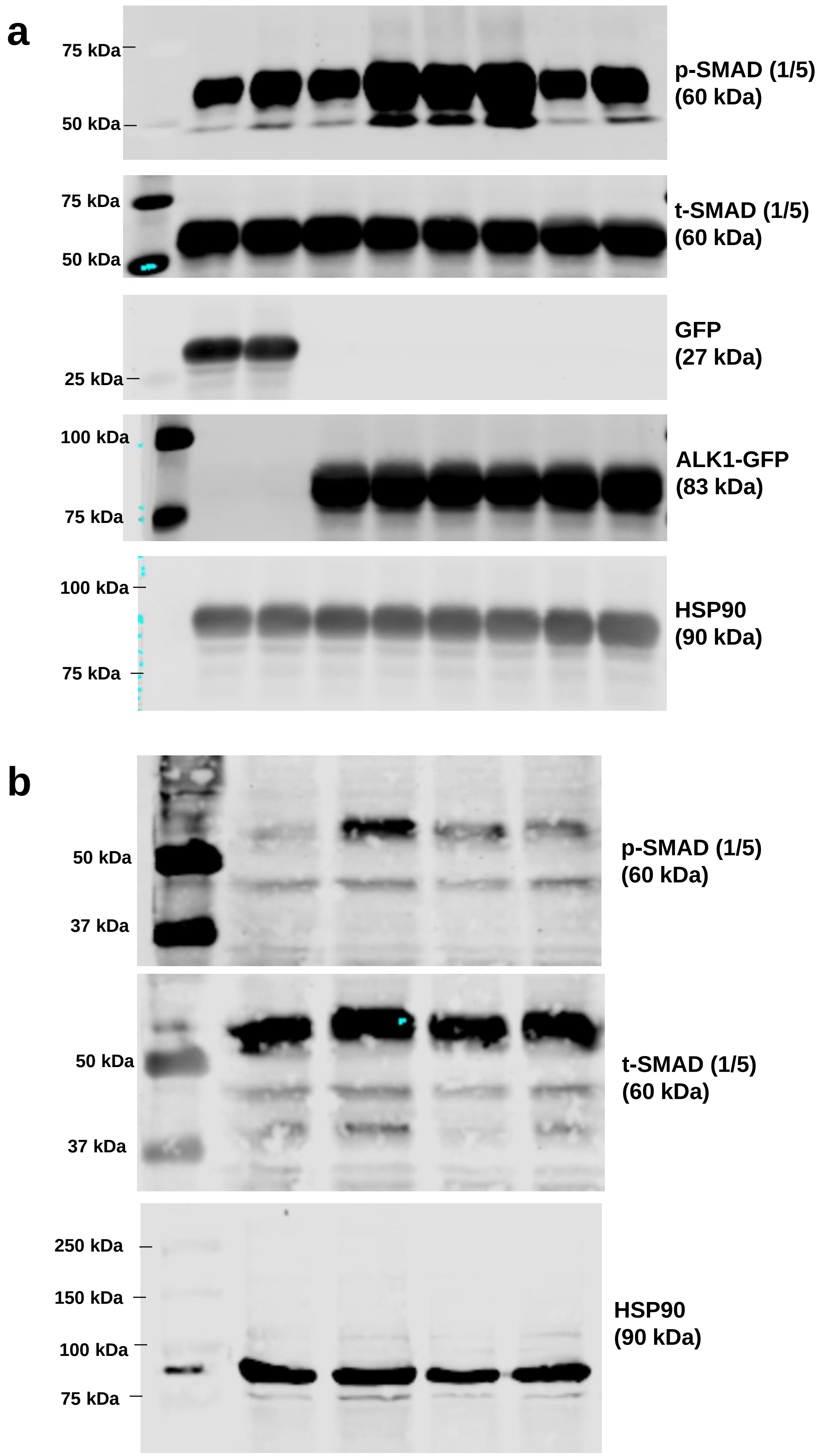

Supplementary Fig. 10. Original Western Blots

a) Original gel scans for Fig. 4c. b) Original gel scans for Fig. 4e.

Supplementary Figure 11

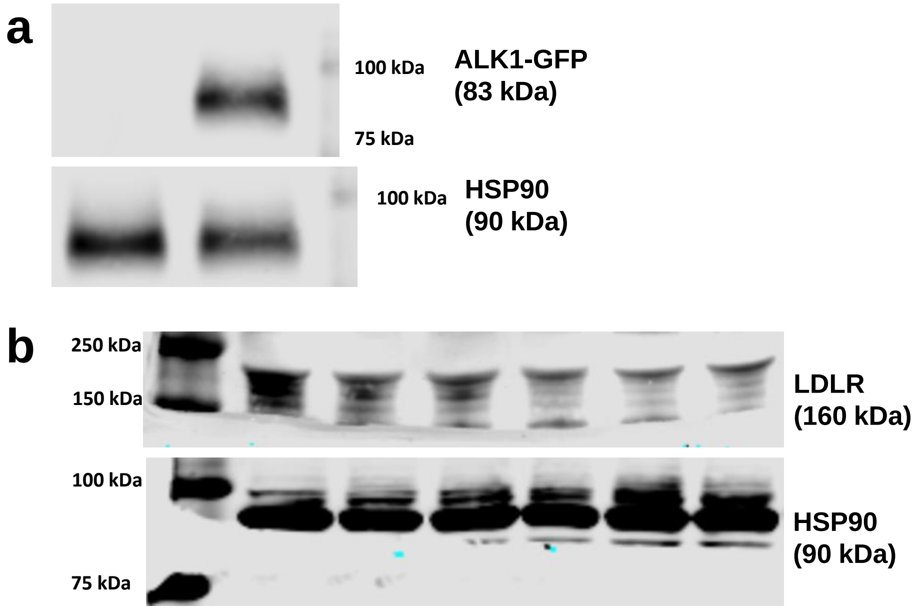

Supplementary Fig. 11. Original Western Blots

a) Original gel scans for Fig. 5a. b) Original gel scans for Sup. Fig. 1b.

Supplementary Figure 12

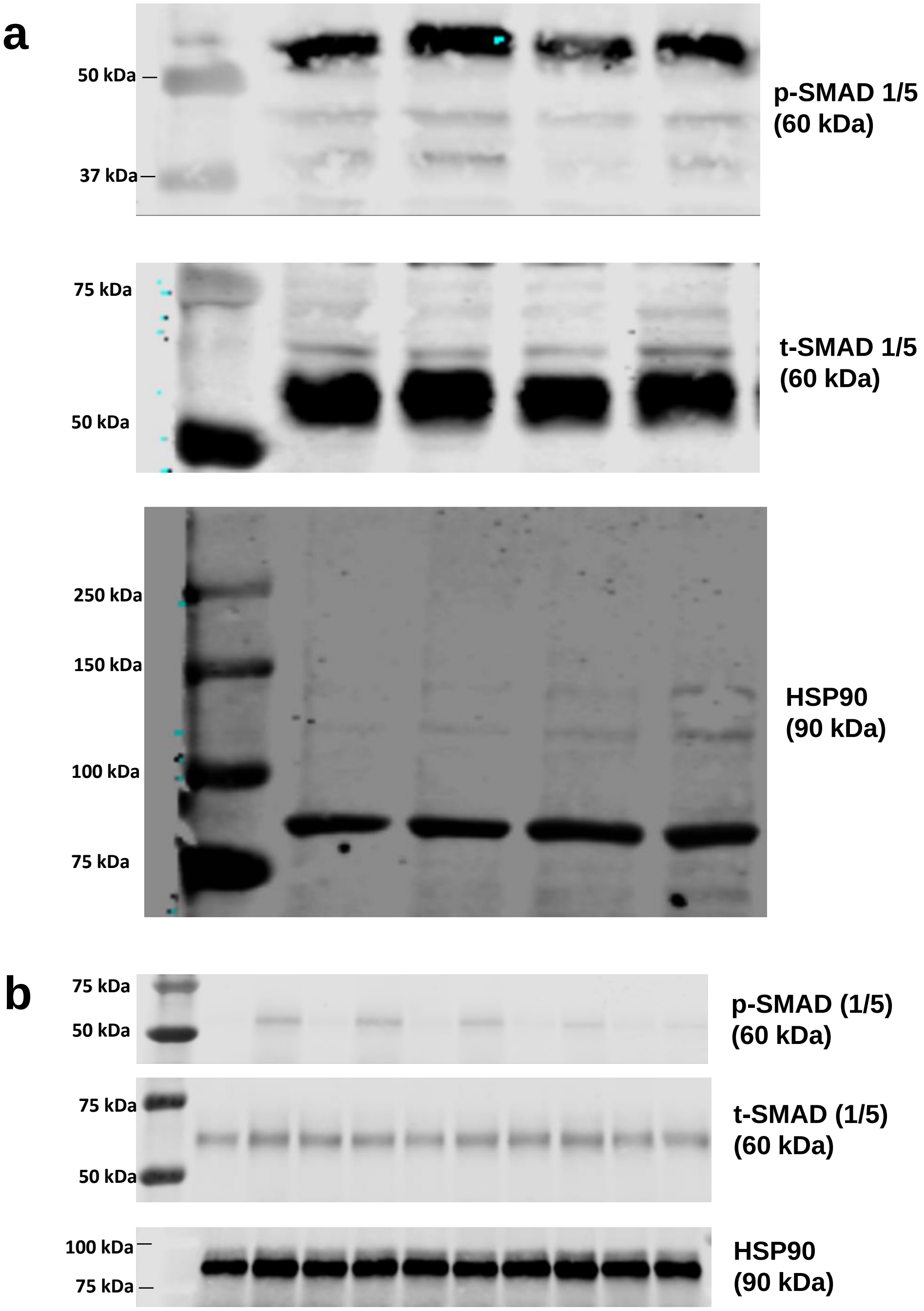

Supplementary Fig. 12. Original Western Blot
a) Original gel scans for Sup. Fig. 6c. b) Original gel scans for Sup. Fig. 9e.

Supplementary Table 1

Network 1	Network 2	Network 3
Hereditary Disorder, Metabolic Disease, Neurological Disease	Carbohydrate Metabolism, Lipid Metabolism, Small Molecule Biochemistry	Cancer, Carbohydrate Metabolism, Cell Cycle
**ACVRL1**, Akt, **ANGPT4**, **ATP6V1C1**, caspase, **CX3CR1**, **ENDOG**, ERK, ERK1/2, **FXYD3**, Gpcr, GPR111, GPR144, GPR152, GPR162, GPR174, **GPR182**, Insulin, **LIF**, **MAPK6**, **NAPA**, NFkB (complex), P38 **MAPK6**, **PSEN2**, **RGR**, **RHBDD3**, RXFP3, **SDC1**, Secretase gamma, **SLC38A3**, **SRP72**, **SYT9**, **TRPC6**, Vegf, VN1R2	**AP2M1**, **ARHGAP9**, ARL17A, ATG4D, **C17orf59**, CCDC94, **CHMP4B**, DYNLL1, FAHD2A, FAM216A, GDAP2, **GJA3**, **GSKIP**, **HLA-QB1**, HNF4A, **MIEF2**, **MRGPRE**, MTRF1L, ORMDL2, PAX6, phosphatidylserin, **PLLP**, RNF44, SLC35D1, SLC7A6OS, SNX11, **SUPT5H**, **SYS1**, **SYTL2**, TMUB2, **TREML2**, TTC26, UBC, ZNF222, ZNF586	IL2, **TSPAN32**

Supplementary Table 1. Qiagen's Ingenuity Pathway Analysis.
Analysis of the 34 gene hits revealed 3 distinct networks. Representation in a table format. Genes in bold were found in the screen and genes in red cluster in the vesicular trafficking pathway.

Supplementary Table 2

gene	ordering number	company	concentration
**control siRNA**	D-001220-01	Dharmacon	20 nM
**scamble control siRNA**	1027310	Qiagen	20 nM
**ALK1 siRNA (human)**	D-005302-06	Dharmacon	20 nM
**ALK1 siRNA (murine)**	M-043004-01	Dharmacon	20 nM
**DNM2 siRNA (human)**	customized<br>target sequence:<br>5'-AACATGCCGAGTTTTTGCACT-3'	Qiagen	20 nM
**LDLR siRNA (human)**	4392420 / ID: s224006	Ambion	20 nM
**LDLR shRNAs lentiviral knockdown (human)**	TRCN0000056517<br>5′-<br>CCGGACAGAGGATGAGGTCCACATTCTCGAGAAT<br>GTGGACCTCATCCTCTGTTTTTTG-3′<br><br>TRCN0000262146<br>5′-<br>CCGGGGGCGACAGATGCGAAAGAAACTCGAGTTT<br>CTTTCGCATCTGTCGCCCTTTTTG-3′<br><br>TRCN0000262148<br>5′-<br>CCGGACATCAACAGCATCAACTTTGCTCGAGCAA<br>AGTTGATGCTGTTGATGTTTTTTG-3′<br><br>TRCN0000262149<br>5′-<br>CCGGATGGAAGAACTGGCGGCTTAACTCGAGTTA<br>AGCCGCCAGTTCTTCCATTTTTTG-3′<br><br>TRCN0000282124<br>5′-<br>CCGGGATGAAGTTGGCTGCGTTAATCTCGAGATT<br>AACGCAGCCAACTTCATCTTTTTG-3′	Sigma-Aldrich	n/a
**NPC2 siRNA (human)**	M-017216-00	Dharmacon	20 nM

Supplementary Tab. 2. siRNA and shRNAs used in the study.

Supplementary Table 3

protein	ordering number	company	working concentration
Western Blot			
**β-actin**	sc-47778	Santa Cruz	1 : 1,000
**HSP90**	sc-13119	Santa Cruz	1 : 1,000
**GFP**	sc-8664	Santa Cruz	1 : 1,000
**LDLR**	10007665	Cayman	1 : 500
**p-SMAD 1/5 (S463/S465)**	9516	Cell Signaling	1 : 500
**t-SMAD 1**	9743	Cell Signaling	1 : 1,000
FACS			
**LDLR**	sc-18823	Santa Cruz	1.5 µg/tube
**mmIgG2b**	sc-3879	Santa Cruz	1.5 µg/tube
**Goat anti-Mouse IgG (H+L) Secondary Antibody, Alexa Fluor® 488 conjugate**	A-11001	Invitrogen	1:500
Immunofluorescence			
**EEA1**	610456	BD Transduction Laboratories	1:200
MLEC isolation			
**M-450 Dynabeads conjugated with sheep anti-rat IgG**	110.07	Dynal Biotech	50 µl beads/6 µl antibody
**Affinity-purified anti-mouse CD31 (PECAM-1) antibody**	553370	Pharmingen	6 µl antibody/3 mice

Supplementary Tab. 3. Antibodies used in the study.

Supplementary Table 4

Cdh5-CreERT2		
forward	5’-GCC TGC ATT ACC GGT CGA TGC AAC GA-3’	
reverse	5'-GTG GCA GAT GGC GCG GCA ACA CCA TT-3'	
amplicon size	700 bp	
PCR program		
step 1	93°C	2:00 min
step 2	93°C	0:30 min
step 3	67°C	0:30 min
step 4	72°C	0:45 min
step 5	go to 2, 35 x	
step 6	72°C	10:00 min
step 7	4°C	forever

LDLR-KO		
P1 (common)	5’-CCA TAT GCA TCC CCA GTC TT-3’	
P2 (WT)	5'-GCG ATG GAT ACA CTC ACT GC-3'	
P3 (Neo)	5’- AAT CCA TCT TGT TCA ATG GCC GAT C-3’	
WT	167 bp	
KO	350 bp	
Het	167 bp + 350 bp	
PCR program		
step 1	95°C	3:00 min
step 2	95°C	0:10 min
step 3	61°C	0:45 min
step 4	72°C	3:00 min
step 5	go to 2, 35 x	
step 6	72°C	10:00 min
step 7	4°C	forever

ALK1 floxed allel		
forward loxP 3	5’-CAG CAC CTA CAT CTT GGG TGG AGA-3’	
reverse loxP 3	5'-ACT GTT CTT CCT CGG AGC CTT GTC-3'	
amplicon size floxed	> 300 bp	
amplicon size unfloxed	187 bp	
forward loxP 6	5’-CCT GGA CAG CGA CTG TAC TAC-3’	
reverse loxP 6	5'-GCC CCA TTG CTC TCC TCA AA-3'	
amplicon size floxed	> 400 bp	
amplicon size unfloxed	356 bp	
By using forward loxP 3 and reverse loxP 6 primers the Δ-band after Cre-recombinase excision can be detected (~ 400 bp).		
PCR program		
step 1	94°C	10:00 min
step 2	94°C	0:30 min
step 3	60°C	0:40 min
step 4	72°C	0:40 min
step 5	go to 2, 34 x	
step 6	72°C	2:00 min
step 7	4°C	forever

Supplementary Tab. 4. Genotyping primers and PCR programs.

Supplementary Table 5

gene	species	primers (5'→3')<br>forward<br>reverse
ALK1	human	CGAGGGATGAACAGTCCTGG<br>GTCATGTCTGAGGCGATGAAG
	murine	GGGCCTTTTGATGCTGTCG<br>TGGCAGAATGGTCTCTTGCAG
DNM2	human	GTTTGTGCTGACTGCCGAGT<br>TTCCAGCTGTCCACGTCTTC
GAPDH	human	CTCTCTGCTCCTCCTGTTCGAC<br>TGAGCGATGTGGCTCGGCT
	murine	AATGTGTCCGTCGTGGATCTGA<br>AGTGTAGCCCAAGATGCCCTTC
HMGCR	human	TGATTGACCTTTCCAGAGCAAG<br>CTAAAATTGCCATTCCACGAGC
INSIG1	human	GCACTGCATTAAACGTGTGG<br>GCAGCACTGAAATGAATGGA
LDLR	human	TCTGCAACATGGCTAGAGACT<br>TCCAAGCATTCGTTGGTCCC
PCSK9	human	GGAGCTGGCCTTGAAGTTGCC<br>ACCGTGGAGGGGTAATCCGC

Supplementary Tab. 5. Primers used for qPCR analyses.

Supplementary References

1. Larrivee B, Prahst C, Gordon E, del Toro R, Mathivet T, Duarte A, Simons M, Eichmann A. Alk1 signaling inhibits angiogenesis by cooperating with the notch pathway. Developmental Cell. 2012;22:489-500

2. Lee HJ, Cho CH, Hwang SJ, Choi HH, Kim KT, Ahn SY, Kim JH, Oh JL, Lee GM, Koh GY. Biological characterization of angiopoietin-3 and angiopoietin-4. FASEB journal : official publication of the Federation of American Societies for Experimental Biology. 2004;18:1200-1208

3. Meng J, Wang J, Lawrence GW, Dolly JO. Molecular components required for resting and stimulated endocytosis of botulinum neurotoxins by glutamatergic and peptidergic neurons. FASEB journal : official publication of the Federation of American Societies for Experimental Biology. 2013

4. Takefuji M, Asano H, Mori K, Amano M, Kato K, Watanabe T, Morita Y, Katsumi A, Itoh T, Takenawa T, Hirashiki A, Izawa H, Nagata K, Hirayama H, Takatsu F, Naoe T, Yokota M, Kaibuchi K. Mutation of arhgap9 in patients with coronary spastic angina. Journal of human genetics. 2010;55:42-49

5. Feng S, Deng L, Chen W, Shao J, Xu G, Li YP. Atp6v1c1 is an essential component of the osteoclast proton pump and in f-actin ring formation in osteoclasts. The Biochemical journal. 2009;417:195-203

6. Hundsrucker C, Skroblin P, Christian F, Zenn HM, Popara V, Joshi M, Eichhorst J, Wiesner B, Herberg FW, Reif B, Rosenthal W, Klussmann E. Glycogen synthase kinase 3beta interaction protein functions as an a-kinase anchoring protein. J Biol Chem. 2010;285:5507-5521

7.The Human Protein Atlas.
Uhlen M, Fagerberg L, Hallstrom BM, Lindskog C, Oksvold P, Mardinoglu A, Sivertsson A, Kampf C, Sjostedt E, Asplund A, Olsson I, Edlund K, Lundberg E, Navani S, Szigyarto CA, Odeberg J, Djureinovic D, Takanen JO, Hober S, Alm T, Edqvist PH, Berling H, Tegel H, Mulder J, Rockberg J, Nilsson P, Schwenk JM, Hamsten M, von Feilitzen K, Forsberg M, Persson L, Johansson F, Zwahlen M, von Heijne G, Nielsen J, Ponten F. Proteomics. Tissue-based map of the human proteome. Science. 2015;347:1260419

8. Martinelli N, Hartlieb B, Usami Y, Sabin C, Dordor A, Miguet N, Avilov SV, Ribeiro EA, Jr., Gottlinger H, Weissenhorn W. Cc2d1a is a regulator of escrt-iii chmp4b. J Mol Biol. 2012;419:75-88

9. Hochheiser K, Heuser C, Krause TA, Teteris S, Ilias A, Weisheit C, Hoss F, Tittel AP, Knolle PA, Panzer U, Engel DR, Tharaux PL, Kurts C. Exclusive cx3cr1 dependence of kidney dcs impacts glomerulonephritis progression. The Journal of clinical investigation. 2013;123:4242-4254

10. McDermott-Roe C, Ye J, Ahmed R, Sun XM, Serafin A, Ware J, Bottolo L, Muckett P, Canas X, Zhang J, Rowe GC, Buchan R, Lu H, Braithwaite A, Mancini M, Hauton D, Marti R, Garcia-Arumi E, Hubner N, Jacob H, Serikawa T, Zidek V, Papousek F, Kolar F, Cardona M, Ruiz-Meana M, Garcia-Dorado D, Comella JX, Felkin LE, Barton PJ, Arany Z, Pravenec M, Petretto E, Sanchis D, Cook SA. Endonuclease g is a novel determinant of cardiac hypertrophy and mitochondrial function. Nature. 2011;478:114-118

11. Bibert S, Roy S, Schaer D, Felley-Bosco E, Geering K. Structural and functional properties of two human fxyd3 (mat-8) isoforms. J Biol Chem. 2006;281:39142-39151

12. Li L, Cheng C, Xia CH, White TW, Fletcher DA, Gong X. Connexin mediated cataract prevention in mice. PLoS One. 2010;5

13. Xiao L, Harrell JC, Perou CM, Dudley AC. Identification of a stable molecular signature in mammary tumor endothelial cells that persists in vitro. Angiogenesis. 2014;17:511-518

14. Griffioen M, van der Meijden ED, Slager EH, Honders MW, Rutten CE, van Luxemburg-Heijs SA, von dem Borne PA, van Rood JJ, Willemze R, Falkenburg JH. Identification of phosphatidylinositol 4-kinase type ii beta as hla class ii-restricted target in graft versus leukemia reactivity. Proc Natl Acad Sci U S A. 2008;105:3837-3842

15. Liu SC, Tsang NM, Chiang WC, Chang KP, Hsueh C, Liang Y, Juang JL, Chow KP, Chang YS. Leukemia inhibitory factor promotes nasopharyngeal carcinoma progression and radioresistance. The Journal of clinical investigation. 2013;123:5269-5283

16. Seternes OM, Mikalsen T, Johansen B, Michaelsen E, Armstrong CG, Morrice NA, Turgeon B, Meloche S, Moens U, Keyse SM. Activation of mk5/prak by the atypical map kinase erk3 defines a novel signal transduction pathway. The EMBO journal. 2004;23:4780-4791

17.Cox PJ, Pitcher T, Trim SA, Bell CH, Qin W, Kinloch RA. The effect of deletion of the orphan g - protein coupled receptor (gpcr) gene mrge on pain-like behaviours in mice. Molecular pain. 2008;4:2

Supplementary References *(continuation)*

18. Ferland RJ, Batiz LF, Neal J, Lian G, Bundock E, Lu J, Hsiao YC, Diamond R, Mei D, Banham AH, Brown PJ, Vanderburg CR, Joseph J, Hecht JL, Folkerth R, Guerrini R, Walsh CA, Rodriguez EM, Sheen VL. Disruption of neural progenitors along the ventricular and subventricular zones in periventricular heterotopia. Human molecular genetics. 2009;18:497-516

19.Miller AD, Bergholz U, Ziegler M, Stocking C. Identification of the myelin protein plasmolipin as the cell entry receptor for mus caroli endogenous retrovirus. J Virol. 2008;82:6862-6868

20.Rivabene R, Visentin S, Piscopo P, De Nuccio C, Crestini A, Svetoni F, Rosa P, Confaloni A. Thapsigargin affects presenilin-2 but not presenilin-1 regulation in sk-n-be cells. Experimental biology and medicine. 2013

21.Ksantini M, Senechal A, Bocquet B, Meunier I, Brabet P, Hamel CP. Screening genes of the visual cycle rgr, rbp1 and rbp3 identifies rare sequence variations. Ophthalmic genetics. 2010;31:200-204

22.. Liu J, Liu S, Xia M, Xu S, Wang C, Bao Y, Jiang M, Wu Y, Xu T, Cao X. Rhomboid domain-containing protein 3 is a negative regulator of tlr3-triggered natural killer cell activation. Proc Natl Acad Sci U S A. 2013;110:7814-7819

23.Bathgate RA, Halls ML, van der Westhuizen ET, Callander GE, Kocan M, Summers RJ. Relaxin family peptides and their receptors. Physiological reviews. 2013;93:405-480

24.Szatmari T, Dobra K. The role of syndecan-1 in cellular signaling and its effects on heparan sulfate biosynthesis in mesenchymal tumors. Frontiersoncology. 2013;3:310

25.Jenstad M, Chaudhry FA. The amino acid transporters of the glutamate/gaba-glutamine cycle and their impact on insulin and glucagon secretion. Frontiers in endocrinology. 2013;4:199

26. Liu T, Yu R, Jin S-B, Han L, Lendahl U, Zhao J, Nistér M. The mitochondrial elongation factors mief1 and mief2 exert partially distinct functions in mitochondrial dynamics. Experimental Cell Research. 2013;319:2893-2904

27.Kirwan M, Walne Amanda J, Plagnol V, Velangi M, Ho A, Hossain U, Vulliamy T, Dokal I. Exome sequencing identifies autosomal-dominant srp72 mutations associated with familial aplasia and myelodysplasia. The American Journal of Human Genetics. 2012;90:888-892

28.Wier AD, Mayekar MK, Heroux A, Arndt KM, VanDemark AP. Structural basis for spt5-mediated recruitment of the paf1 complex to chromatin. Proc Natl Acad Sci U S A. 2013;110:17290-17295

29.Setty SR, Strochlic TI, Tong AH, Boone C, Burd CG. Golgi targeting of arf-like gtpase arl3p requires its nalpha-acetylation and the integral membrane protein sys1p. Nature cell biology. 2004;6:414-419

30.Iezzi M, Eliasson L, Fukuda M, Wollheim CB. Adenovirus-mediated silencing of synaptotagmin 9 inhibits ca2+-dependent insulin secretion in islets. FEBS Letters. 2005;579:5241-5246

31.Ménasché G, Ménager MM, Lefebvre JM, Deutsch E, Athman R, Lambert N, Mahlaoui N, Court M, Garin J, Fischer A, de Saint Basile G. A newly identified isoform of slp2a associates with rab27a in cytotoxic t cells and participates to cytotoxic granule secretion. Blood. 2008;112:5052-5062

32.Allcock RJ, Barrow AD, Forbes S, Beck S, Trowsdale J. The human trem gene cluster at 6p21.1 encodes both activating and inhibitory single igv domain receptors and includes nkp44. European journal of immunology. 2003;33:567-577

33.Seo K, Rainer PP, Shalkey Hahn V, Lee D-i, Jo S-H, Andersen A, Liu T, Xu X, Willette RN, Lepore JJ, Marino JP, Birnbaumer L, Schnackenberg CG, Kass DA. Combined trpc3 and trpc6 blockade by selective small-molecule or genetic deletion inhibits pathological cardiac hypertrophy. Proceedings of the National Academy of Sciences. 2014

34.Gartlan KH, Belz GT, Tarrant JM, Minigo G, Katsara M, Sheng K-C, Sofi M, van Spriel AB, Apostolopoulos V, Plebanski M, Robb L, Wright MD. A complementary role for the tetraspanins cd37 and tssc6 in cellular immunity. The Journal of Immunology. 2010;185:3158-3166
